# An efficient synthesis of RNA containing GS-441524: the nucleoside precursor of remdesivir†

**DOI:** 10.1039/d1ra06589k

**Published:** 2021-09-22

**Authors:** Ramkumar Moorthy, Samantha A. Kennelly, Deborah J. Rodriguez, Daniel A. Harki

**Affiliations:** Department of Medicinal Chemistry, University of Minnesota 2231 6^th^ Street S.E. Minneapolis MN 55455 USA daharki@umn.edu

## Abstract

Remdesivir is an antiviral nucleoside phosphoramidate with activity against multiple viruses, including SARS-CoV-2. To enable studies of viral polymerases with RNA containing remdesivir, we report an efficient synthesis of a phosphoramidite of GS-441524, the nucleoside precursor of remdesivir, and its incorporation into RNA using automated solid-phase RNA synthesis.

## Abstract 2

Remdesivir in RNA: An efficient synthesis of a GS-441524-containing phosphoramidite for solid-phase RNA synthesis is reported.

## Graphical abstract

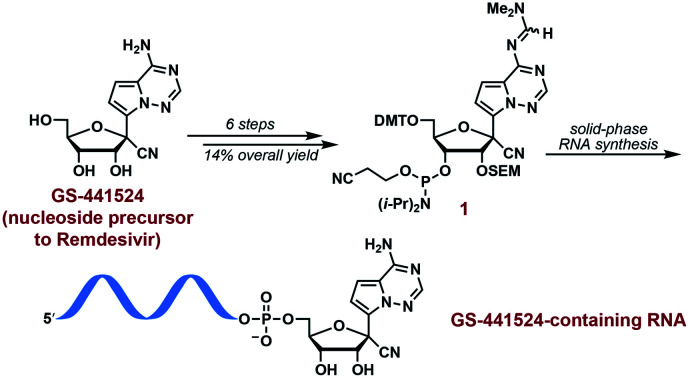

Remdesivir is a 1′-cyano-substituted, 4-aza-7,9-dideazaadenosine phosphoramidate prodrug that displays broad-spectrum antiviral activity against viruses such as hepatitis C (HCV), yellow fever (YFV), dengue-2 (DENV-2), influenza A, parainfluenza 3, Ebola virus (EBOV) and SARS-CoV (Fig. 1).^1^ Currently, remdesivir is the only nucleoside drug approved by the US Food and Drug Administration to combat COVID-19.^2^ Remdesivir is metabolized intracellularly to its 5′-monophosphate and then further bio-transformed to its active metabolite, the 5′-triphosphate.^3^ Viral RNA-dependent RNA polymerases (RdRp) mis-incorporate the resulting nucleotide, which stalls RNA synthesis resulting in antiviral effects.^4^ This promising antiviral activity has prompted studies of viral polymerases with RNA-incorporated drug, necessitating methods to synthesize RNA containing the nucleoside precursor of remdesivir, GS-441524 (Fig. 1). Recently, a GS-441524 phosphoramidite synthesis was reported and that compound was subsequently used in solid-phase RNA synthesis to enable mechanistic studies of SARS-CoV-2 RdRp stalling.^5^ Here, we report a concise and high-yielding synthesis of an alternative GS-441524-containing phosphoramidite, 1, which is prepared in 14% overall yield in 6 steps from GS-441524.

**Fig. 1:**
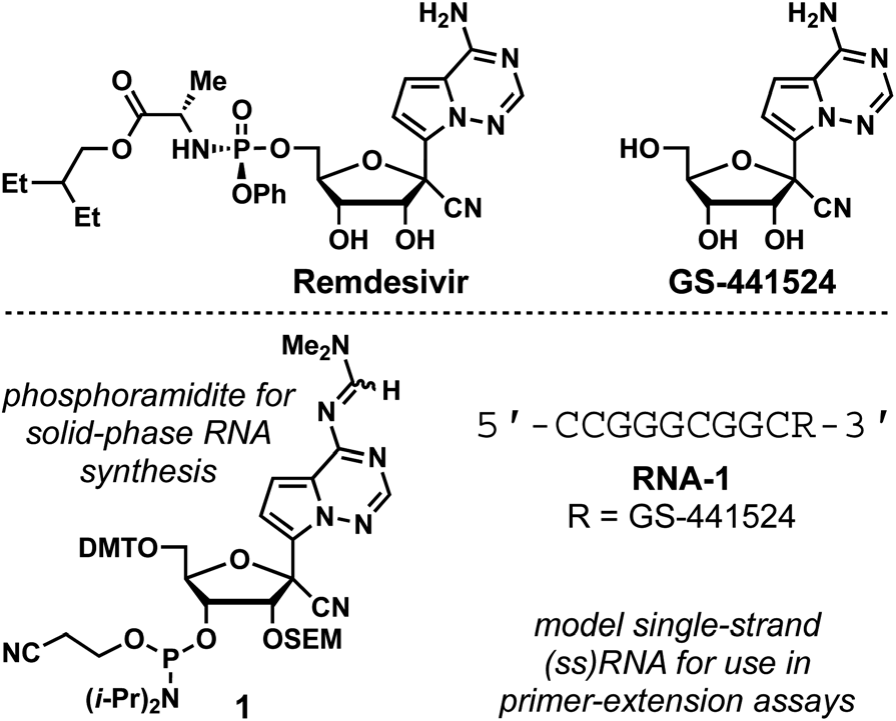
Structures of remdesivir, GS-441524, phosphoramidite 1, and RNA-1.

We initiated the synthesis of phosphoramidite 1 by attempting to protect the primary exocyclic amine of GS-441524 with a benzoyl group using the Jones transient *N*-benzoylation method (trimethylsilyl chloride, benzoyl chloride, pyridine and NH_4_OH), which resulted in decomposed product.^6^ Therefore, we altered the order of protecting group installation. The 3′- and 5′-alcohols were protected using di(*tert*-butyl)chlorosilane^7^ to yield 2 in 86% yield (Scheme 1). Of note, this protecting group can be selectively removed under mild acidic conditions^8,9^ without affecting other fluoride-labile groups present at the 2′-OH (*e.g.*, SEM, TBS, or TOM). We next turned our attention to protecting the exocyclic amine with dimethyl-formamidine due to constrains imposed by the free 2′-OH in using other protecting groups. Protection of the amine of 2 was optimized by reacting 2 with *N*,*N*-dimethylformamide dimethyl acetal under various reaction conditions (Table 1).

**Scheme 1:**
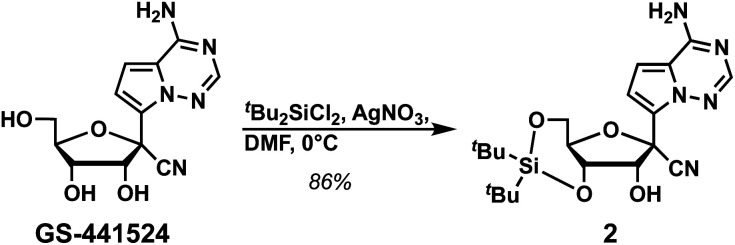
Synthesis of compound 2.

**Table 1:** Optimization of exocyclic amine 2 protection using *N*,*N*-dimethylformamide dimethyl acetal^a^

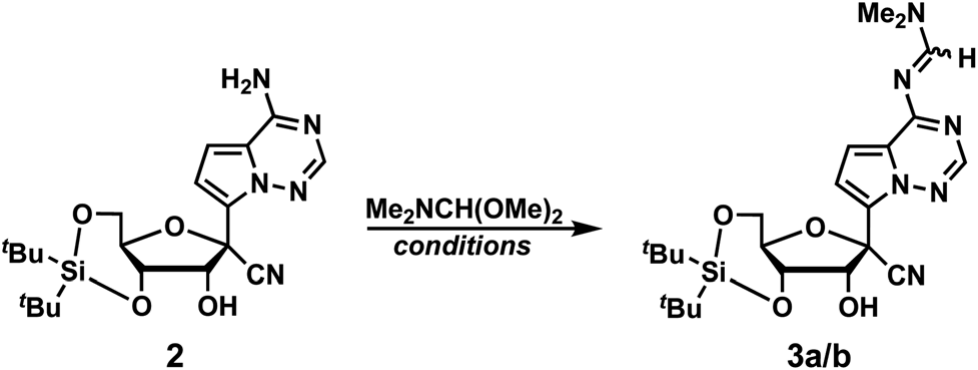				
Entry	Temp (°C)	Time (h)	Solvent	Yield^e^ (%)
1	RT	48	DMF	66
2	65	24	DMF	58
3^b^	65	24	DMF	57
4^c^	65	24	DMF	55
5^d^	65	1	DMF	59
6	65	24	DMA	52
7	65	24	Pyridine	57
8^f^	65	24	—	71

a Unless otherwise stated, the reactions were performed on a 0.10 mmol scale using 2 (1.0 equiv.) and *N*,*N*-dimethylformamide dimethyl acetal (4.0 equiv.) in the corresponding solvent (1.0 mL organic solvent).

b Reaction performed with MS 4 Å (50 mg).

c Reaction performed with MgSO_4_ (50 mg).

d Reaction was performed in a microwave synthesizer at 200 W, 17 PSI.

e Yield represents a mixture of diastereomers (for entries 1–7 the reaction resulted in 3a as the major diastereomer and 3b as the minor diastereomer).

f Reaction yielded 3b as the only diastereomer. DMF: *N*,*N*-dimethyl formamide; DMA: *N*,*N*-dimethyl formamide acetal. Reactions were performed in duplicate and yields shown are an average. Note: 3a and 3b are separable diastereomers; stereochemical assignments of the imines of 3a and 3b were not conducted since both protecting groups are ultimately cleaved.

Initially, the reaction was performed in DMF at room temperature for 48 hours yielding imine diastereomers 3a and 3b in 66% yield. The *E*/*Z* stereochemical identities of 3a and 3b were not determined due to a lack of diagnostic NMR resonances and the inconsequential nature of the olefin geometry: the imine protecting groups are ultimately cleaved. Of note, diastereomer 3a has the higher chromatographic R_f_ compared to 3b. To improve reaction efficiency, the solution was heated at 65 °C for 24 hours, which resulted in a similar yield of product formation (58%). For imine condensation, removal of water drives the reaction towards product formation, therefore the reaction was performed with additives such as 4 Å molecular sieves and MgSO_4_ at 65 °C. However, neither condition improved the reaction yield (entries 3 and 4, Table 1). We next investigated microwave conditions (200 W, 17 PSI) with heating to 65 °C for 1 hour, which resulted in similar yields to reactions performed at room temperature or at 65 °C (entry 5, Table 1). Switching solvent to DMA or pyridine resulted in a lower or similar yield compared to DMF (entries 6, and 7, Table 1). Interestingly, solvent-free conditions resulted in a slight improvement of product formation compared to reactions performed with solvent (entry 8, Table 1). However, the neat condition did not result in the full conversion, and we were unable to purify product from the starting material. Since these studies resulted in no significant improvement in yield, we used conditions from entry 1 (Table 1) with pure diastereomer 3a to continue the synthesis of phosphoramidite 1.

We next investigated conditions to protect the 2′-alcohol of 3a. Although a variety of 2′-alcohol protecting groups have been successfully utilized in phosphoramidite synthesis, including base-labile,^10^ acid-labile,^11^ fluoride-labile,^12^ reductively removable,^13^ and photo-labile groups,^14^ we elected to utilize a fluoride-labile protecting group and initially investigated the 2′-OTBS group. However, to our surprise, we found that the typical reaction conditions to install TBS groups (*e.g.*, TBSCl, imidazole, DMAP and DMF at room temperature for 48 hours) resulted in no reaction. Addition of AgNO_3_ in pyridine as an activator yielded 5a in 50% yield. However, we recognized multiple issues with the 2′-OTBS protection based on prior literature, including instability in ammonia solutions,^15^ chain cleavage,^16^ silyl migration to the 3′-alcohol,^17^ and longer coupling times during oligonucleotide synthesis due to steric bulk.^18^ Hence, we switched to trimethylsilyl-ethoxymethyl (SEM) as a 2′-alcohol protecting group, which resulted in a higher yield (5b, 74%). Next, the 3′,5′-*O*-di(*tert*-butyl)silyl group was selectively deprotected using Olah's reagent to afford 6 in 82% yield. Finally, 1 was prepared in 40% yield over two steps by protecting the 5′-OH with 4,4′-dimethoxytrityl chloride (DMTCl), followed by the reaction with 2-cyanoethyl *N*,*N*-diisopropylchloro-phosphoramidite. Taken together, GS-441524 phosphoramidite 1 was synthesized in 6 linear steps with an overall yield of 14% (Scheme 2). A notable advantage of our work in comparison to the published GS-441524 phosphoramidite synthesis is the lack of a requirement for isomerization of a 3′-OTBS group to install the requisite 2′-OTBS protecting group, which occurs in modest yield, and concomitant separation of 2′-OTBS and 3′-OTBS isomers.^5^

**Scheme 2:**
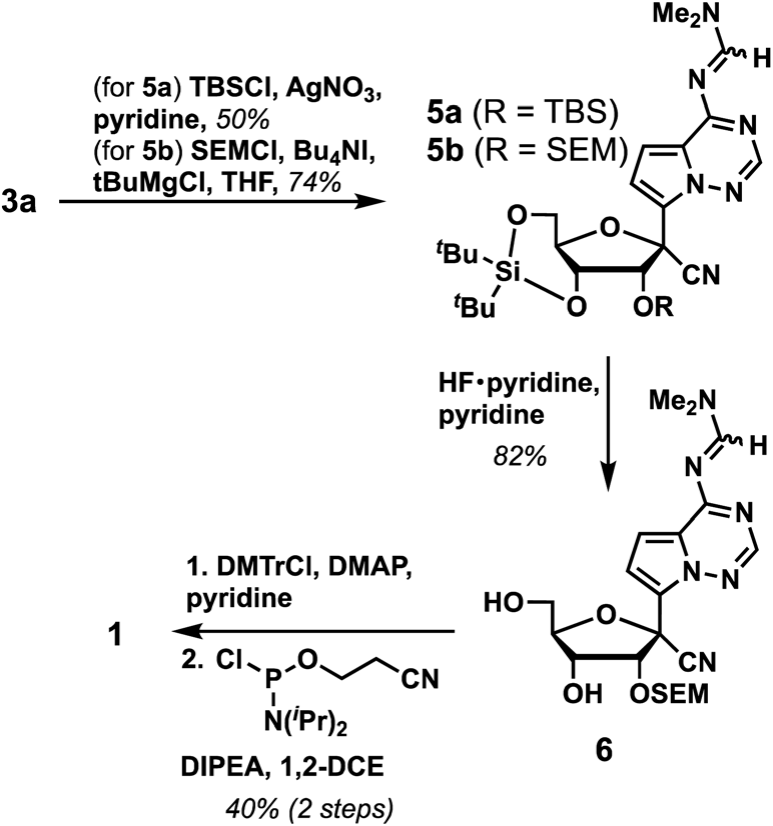
Synthesis of GS-441524 phosphoramidite 1.

We further investigated approaches to improve the yield of the first two steps of the synthesis of 1. Accordingly, we found that protecting the exocyclic amine of GS-441524 with *N*,*N*-dimethylformamide dimethyl acetal in DMF in the first step resulted in formation of 4 in 66% yield. Changing the solvent from DMF to pyridine resulted in a higher yield (76%; Scheme 3). Next, the 3′- and 5′-alcohols were protected using di(*tert*-butyl)chlorosilane in high yield (85%, see ESI:†3b). To our delight, switching the protection sequence improved the overall yield (65% for two steps) compared to the previous strategy (Scheme 1 and Table 1, 56% for two steps), as well as yielded single diastereomer 3b, which simplified chromatographic separation. This modified strategy can be utilized for the synthesis of GS-441524 phosphoramidite 1 as shown in Scheme 2.

**Scheme 3:**
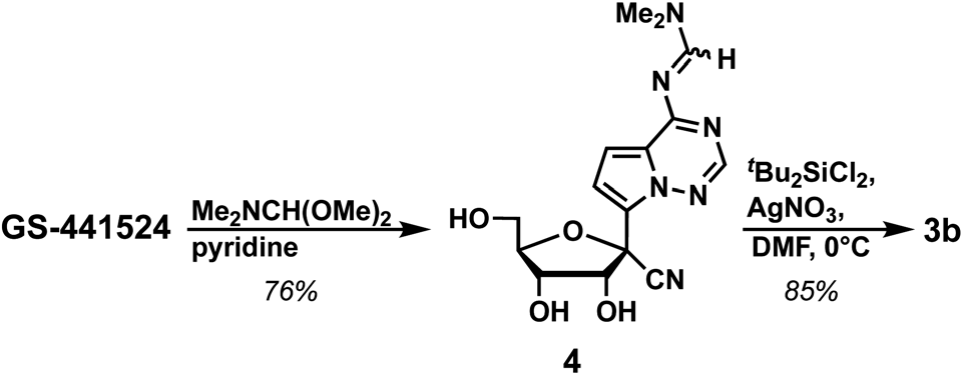
Synthesis of 3b.

With phosphoramidite 1 in-hand, we utilized standard RNA synthesis methods to prepare 5′-CCGGGCGGCR-3′ (RNA-1), where R is GS-441524. RNA-1 was synthesized in reasonable yield (135 nmol from a 1000 nmol-scale synthesis) and high purity (91% following HPLC purification; Fig. 2).

**Fig. 2:**
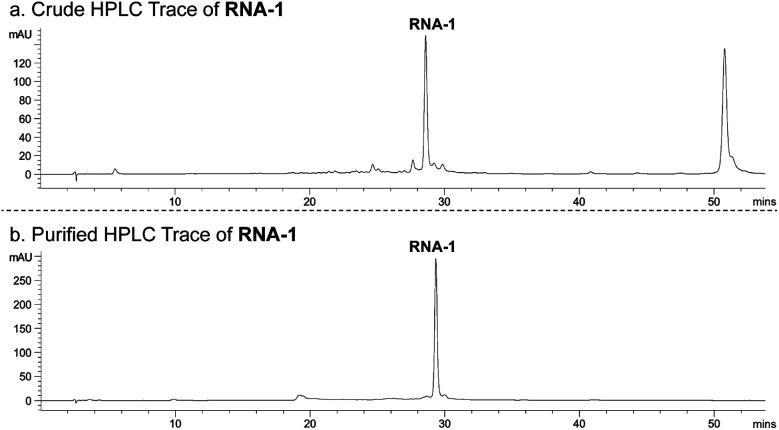
HPLC chromatogram of (a) crude RNA-1 and (b) HPLC-purified RNA-1 (monitored at 260 nm).

In conclusion, we have developed an efficient synthesis of a GS-441524 phosphoramidite that requires six linear steps and results in an overall yield of 14% starting from the GS-441524 nucleoside. GS-441524-containing phosphoramidite 1 was successfully used in automated solid-phase RNA synthesis to demonstrate the utility of our novel phosphoramidite. Phosphoramidite 1 thus serves as a useful new reagent for preparing RNA containing the promising antiviral drug remdesivir.

## Author contributions

RM and DAH designed the phosphoramidite synthesis; RM and DJR synthesized compounds. SAK synthesized the RNA. RM, SAK and DAH wrote the manuscript, and all authors agree to its submission.

## Conflicts of interest

The authors declare no conflicts of interest.

## Supplementary Material

RA-011-D1RA06589K-s001
