# Supplementary material for: An efficient synthesis of RNA containing GS-441524: the nucleoside precursor of remdesivir
Source: RSC Adv. 2021 Sep 22;11(50):31373–6. doi: 10.1039/d1ra06589k (PMC9041347; doi:10.1039/d1ra06589k)
Supplement: RA-011-D1RA06589K-s001 [file RA-011-D1RA06589K-s001.pdf]

Electronic Supplementary Material (ESI) for RSC Advances.
This journal is © The Royal Society of Chemistry 2021

Supporting Information

An Efficient Synthesis of RNA Containing GS-441524: The Nucleoside Precursor of Remdesivir

Ramkumar Moorthy, Samantha A. Kennelly, Deborah J. Rodriguez, and Daniel A. Harki*

Department of Medicinal Chemistry, University of Minnesota,
2231 6th Street S.E., Minneapolis, MN, 55455, USA

Table of Contents

I.	General Reaction Conditions	S2
II.	Synthesis of the GS-441524 Phosphoramidite	S3
III.	Protecting Group Studies Towards the Synthesis of the GS-441524 Phosphoramidite	S7
IV.	Preparation of the GS-441524-Containing RNA	S8
V.	Molar Extinction Coefficient Calculation of Remdesivir	S10
VI.	NMR Spectroscopic Data	S11
VII.	LC-MS Trace and Mass Spectra of RNA-1	S30
VII.	References	S31

I. General Reaction Conditions. All commercial chemicals were used as received. Reaction solvents acetonitrile (CH_3CN), dichloromethane (CH_2Cl_2), and tetrahydrofuran (THF) were dried by passage over a column of activated alumina using a solvent purification system (MBraun). Dimethylformamide (DMF) was dried by passage over a column of molecular sieves using a solvent purification system (MBraun). Pyridine, *N,N*-dimethylacetamide (DMA), 2-(trimethylsilyl)ethoxymethyl chloride (SEM-Cl), *tert*-butylmagnesium chloride solution (^{t}BuMgCl, 1M solution in THF), hydrogen fluoride pyridine complex, and 1,2-dichloroethene (1,2-DCE) where obtained from Sigma Aldrich. (2*R*,3*R*,4*S*,5*R*)-2-(4-aminopyrrolo[2,1-*f*][1,2,4]triazin-7-yl)-3,4-dihydroxy-5-(hydroxymethyl)tetrahydrofuran-2-carbonitrile (**GS-441524**) was obtained from Ontario Chemicals Inc. *N*,*N*-dimethylformamide dimethyl acetal was obtained from Acros Organics. *N*,*N*-diisopropylethylamine (DIPEA) was distilled from calcium hydride and stored with 4 Å molecular sieves under argon until use. Synthesized compounds were purified on a Teledyne-Isco Combiflash Rf-200 instrument using Redisep Rf High Performance silica gel columns from Teledyne-Isco. ^{1}H NMR (500 MHz), ^{13}C NMR (126 MHz), and ^{31}P NMR (202 MHz) spectra were recorded on a Bruker Avance NMR spectrometer (500 MHz) at room temperature. NMR chemical shifts (δ) are recorded relative to TMS (0.05% v/v; δ = 0.0) for ^{1}H NMR and the solvent signal for ^{13}C NMR (δ = 77.0 for $CDCl_3$, and δ = 39.7 for $(CD_3)_2SO$). High-resolution mass spectra were obtained at the Analytical Biochemistry Core Facility at the University of Minnesota Masonic Cancer Center using an LTP Orbitrap Velos Mass Spectrometer (Thermo Fisher).

II. Synthesis of the GS-441524 Phosphoramidite.

(4aR,6R,7R,7aS)-6-(4-aminopyrrolo[2,1-f][1,2,4]triazin-7-yl)-2,2-di-tert-butyl-7-hydroxytetrahydro-4H-furo[3,2-d][1,3,2]dioxasiline-6-carbonitrile (2):

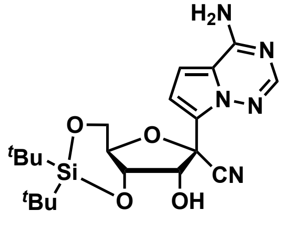

2

To a flame-dried flask under Ar was added **GS-441524** (500 mg, 1.71 mmol), di-*tert*-butyl-di-chlorosilane (0.543 mL, 2.57 mmol), $AgNO_3$ (0.437 g, 2.57 mmol), and DMF (6 mL). The reaction mixture was stirred at 0 ºC for 90 minutes. The reaction was quenched with water (5 mL) and diluted with EtOAc (80 mL). The EtOAc layer was subsequently washed with brine (3 X 50 mL), dried over anhydrous Na_2SO_4, and concentrated *in vacuo*. The crude material was purified by SiO_2 chromatography (gradient 0 – 10% CH_3OH in $CHCl_3$) to give 609 mg (82%) of **2** as a white solid. Yield range (2 runs): 82-90%; average: 86%.

^{1}H NMR (500 MHz, DMSO-d_6): δ 8.46 (s, 1H), 7.42 (d, *J* = 4.5 Hz, 1H), 7.23 (d, *J* = 4.6 Hz, 1H), 7.21 (d, *J* = 4.6 Hz, 1H) 5.19 (app t, *J* = 4.7 Hz, 1H), 4.99 (dd, *J* = 9.2, 5.0 Hz, 1H), 4.71 (td, *J* = 10.1, 5.0 Hz, 1H), 4.49 – 4.41 (m, 2H), 1.51 (s, 9H), 1.46 (s, 9H).

^{13}C NMR (126 MHz, DMSO-d_6): δ 155.5, 148.0, 123.3, 116.4, 116.3, 109.4, 101.1, 81.1, 75.9, 73.9, 73.3, 66.9, 27.1 (3C, ^{t}Butyl), 26.9 (3C, ^{t}Butyl), 22.2, 20.0.

HRMS: $C_{20}H_{30}N_5O_4Si$ Calculated for $[M+H]^+$: 432.2067; Found: 432.2056.

N'-(7-((4aR,6R,7R,7aS)-2,2-di-tert-butyl-6-cyano-7-hydroxytetrahydro-4H-furo[3,2-d][1,3,2]dioxasilin-6-yl)pyrrolo[2,1-f][1,2,4]triazin-4-yl)-N,N-dimethylformimidamide (3a/b):

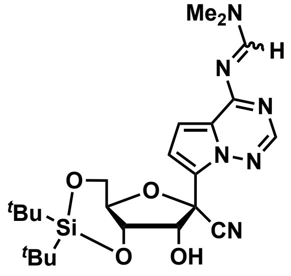

3a/b

To a flame-dried flask under Ar was added **2** (25.1 mg, 0.0579 mmol), *N*,*N*-dimethylformamide dimethyl acetal (27.1 mg, 0.231 mmol), and DMF (1 mL). The reaction mixture was stirred at room temperature for 48 hours. The reaction was quenched with water (2 mL) and was diluted with EtOAc (20 mL). The EtOAc layer was subsequently washed with brine (3 X 20 mL) and dried over sodium sulfate. The drying agent was removed by vacuum filtration and the filtrate was concentrated *in vacuo*. The crude product was purified by SiO_2 chromatography (gradient of 0 – 100% EtOAc in CH_2Cl_2) to give 19 mg (68%) of **3a/b** (mixture of separable isomers) as a yellow solid. Yield range (2 runs): 64-68%; average: 66%.

Isomer 3a: (Rf 0.40; 4:1 EtOAc:CH_2Cl_2)

^{1}H NMR (500 MHz, $CDCl_3$): δ 8.80 (s, 1H), 7.97 (s, 1H), 6.88 (d, *J* = 4.5 Hz, 1H), 6.85 (d, *J* = 4.5 Hz, 1H), 5.71 (s, 1H), 4.78 (d, *J* = 8.7 Hz, 1H), 4.46 – 4.36 (m, 2H), 4.16 – 4.06 (m, 2H), 3.26 (s, 3H), 3.25 (s, 3H), 1.11 (s, 9H), 0.98 (s, 9H).

^{13}C NMR (126 MHz, $CDCl_3$): δ 160.6, 158.1, 147.5, 124.7, 123.1, 116.0, 111.4, 103.4, 79.6, 79.3, 78.7, 75.6, 67.6, 41.8, 35.5, 27.5 (3C, ^{t}Butyl), 27.1 (3C, ^{t}Butyl), 22.7, 20.2.

HRMS: Calculated for $C_{23}H_{35}N_6O_4Si$ $[M+H]^+$: 487.2489; Found: 487.2471.

Isomer 3b:(Rf 0.35; 4:1 EtOAc:CH_2Cl_2)

^{1}**H NMR** (500 MHz, $CDCl_3$): δ 8.77 (s, 1H), 8.02 (s, 1H), 6.84 – 6.81 (m, *J* = 0.9 Hz, 2H), 4.73 (d, *J* = 4.8 Hz, 1H), 4.47 (dd, *J* = 9.2, 4.8 Hz, 1H), 4.31 – 4.19 (m, 2H), 3.98 (app t, *J* = 9.5 Hz, 1H), 3.51 (s, 1H, OH), 3.18 (s, 3H), 3.16 (s, 3H), 0.99 (s, 9H), 0.98 (s, 9H).

^{13}**C NMR** (126 MHz, $CDCl_3$): δ 160.6, 157.7, 147.4, 123.4, 122.8, 115.6, 111.9, 102.5, 82.1, 77.3, 77.0, 76.7, 75.9, 75.1, 73.6, 67.3, 41.5, 35.3, 27.3 (3C, ^{t}Butyl), 27.2 (3C, ^{t}Butyl), 22.6, 20.4.

HRMS: Calculated for $C_{23}H_{35}N_6O_4Si$ $[M+H]^+$: 487.2489; Found: 487.2472.

N'-(7-((4aR,6R,7R,7aR)-2,2-di-tert-butyl-6-cyano-7-((2-(trimethylsilyl)ethoxy)methoxy)tetrahydro-4H-furo[3,2-d][1,3,2]dioxasilin-6-yl)pyrrolo[2,1-f][1,2,4]triazin-4-yl)-N,N-dimethylformimidamide (5b):

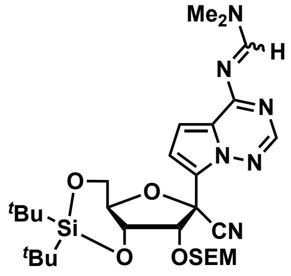

5b

To a flame-dried flask under Ar was added **3a** (250 mg, 0.513 mmol), Bu_4NI (380 mg, 1.02 mmol) and THF (5 mL). To the suspension, ^{t}BuMgCl (1M in THF solution) (0.672 mL, 5.64 mmol) was added and stirred for 10 minutes followed by the dropwise addition of SEM-Cl (0.171 g, 1.02 mmol) dissolved in THF (5 mL). The reaction stirred for 48 hours at room temperature. The reaction was quenched with water (5 mL) and diluted with EtOAc (40 mL). The EtOAc layer was subsequently washed with brine (1 X 50 mL) and dried over anhydrous Na_2SO_4 and then concentrated *in vacuo*. Purification by SiO_2 chromatography (gradient of 0 – 50% EtOAc in CH_2Cl_2) gave 251 mg (79%) of **5b** as a yellow solid. Yield range (2 runs): 68-79%; average: 74%.

^{1}**H NMR (500 MHz, $CDCl_3$):** δ 8.81 (s, 1H), 8.04 (s, 1H), 7.04 (d, *J* = 4.5 Hz, 1H), 6.87 (d, *J* = 4.5 Hz, 1H), 5.46 (d, *J* = 8.8 Hz, 1H), 4.82 (d, *J* = 6.8 Hz, 1H), 4.67 – 4.55 (m, 2H), 4.45 (app t, *J* = 9.2 Hz, 1H), 4.34 (dd, *J* = 9.2, 5.1 Hz, 1H), 4.04 (app t, *J* = 9.9 Hz, 1H), 3.55 (td, *J* = 11.6, 9.7 Hz, 1H), 3.42 (td, *J* = 11.6, 9.7, 1H), 3.23 (s, 3H), 3.20 (s, 3H), 1.09 (s, 9H), 1.04 (s, 9H), 0.72 (td, *J* = 13.6, 11.5 Hz, 1H), 0.59 (td, *J* = 13.6, 11.5 Hz, 1H), -0.08 (s, 9H).

^{13}**C NMR (126 MHz, $CDCl_3$):** δ 160.9, 157.6, 147.5, 124.2, 123.2, 116.8, 113.9, 102.4, 94.2, 80.0, 79.5, 75.5, 67.8, 65.6, 41.5, 35.3, 27.5 (3C, ^{t}Butyl), 27.1 (3C, ^{t}Butyl), 22.7, 20.3, 20.0, 17.8, -1.3 (3C, $-Si(CH_3)_3$).

HRMS: Calculated for $C_{29}H_{49}N_6O_5Si_2$ $[M+H]^+$: 617.3303; Found: 617.3274.

N'-(7-((2R,3R,4R,5R)-2-cyano-4-hydroxy-5-(hydroxymethyl)-3-((2-(trimethylsilyl)ethoxy)-methoxy)tetrahydrofuran-2-yl)pyrrolo[2,1-f][1,2,4]triazin-4-yl)-N,N-dimethylformimidamide (6):

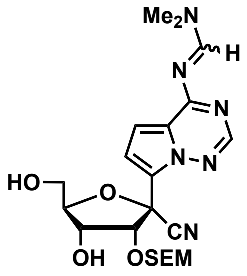

6

To a flame-dried flask under Ar was added **5b** (250 mg, 0.405 mmol) and anhydrous pyridine (5 mL). HF-pyridine (Olah's reagent) (0.115 mL, 4.05 mmol) was added dropwise to the reaction mixture and stirred at room temperature for 1 hour. The reaction was quenched with water (5 mL) and diluted with CH_2Cl_2 (40 mL). The CH_2Cl_2 layer was subsequently washed with brine (1 X 20 mL) and dried over anhydrous Na_2SO_4 followed by concentration *in vacuo*. The crude product was purified by SiO_2 chromatography (gradient of 0 – 40% EtOAc in CH_2Cl_2) to give 152 mg (79%) **6** as a yellow solid. Yield range (2 runs): 79-85%; average: 82%.

^{1}H NMR (500 MHz, CDCl$_3$): δ 8.82 (s, 1H), 8.10 (s, 1H), 7.02 (d, *J* = 4.6 Hz, 1H), 6.89 (d, *J* = 4.6 Hz, 1H), 5.19 (d, *J* = 7.1 Hz, 1H), 4.83 (d, *J* = 7.3 Hz, 1H), 4.68 (d, *J* = 7.3 Hz, 1H), 4.53 – 4.42 (m, 2H), 3.95 (dd, *J* = 12.6, 3.1 Hz, 1H), 3.92 – 3.83 (m, 2H), 3.57 (app q, *J* = 9.2 Hz, 1H), 3.24 (s, 3H), 3.22 (s, 3H), 0.90 (t, *J* = 9.2 Hz, 2H), 0.01 (s, 9H).

^{13}C NMR (126 MHz, CDCl$_3$): δ 160.9, 157.8, 147.6, 124.1, 122.5, 117.1, 114.0, 102.5, 95.4, 85.4, 83.7, 77.6, 74.2, 66.7, 61.6, 41.6, 35.4, 18.0, -1.3 (3C, -Si(CH$_3$)$_3$).

HRMS: Calculated for $C_{21}H_{33}N_6O_5Si$ $[M+H]^+$: 477.2282; Found: 477.2261.

N'-(7-((2R,3R,4R,5R)-5-((bis(4-methoxyphenyl)(phenyl)methoxy)methyl)-2-cyano-4-hydroxy-3-((2-(trimethylsilyl)ethoxy)methoxy)tetrahydrofuran-2-yl)pyrrolo[2,1-f][1,2,4]triazin-4-yl)-N,N-dimethylformimidamide (7):

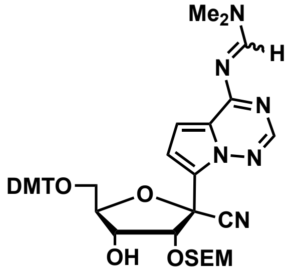

7

To a flame-dried flask under Ar was added 4,4'-dimethoxytrityl chloride (0.159 g, 0.472 mmol), **6** (150 mg, 0.314 mmol), 4-dimethylaminopyridine (42.1 mg, 0.345 mmol) and anhydrous pyridine (2 mL). The reaction mixture was stirred for 18 hours at room temperature. The reaction mixture was concentrated *in vacuo* and purified by chromatography on neutralized SiO_2 (neutralized by washing with 2 column volumes of 10% Et_3N in CH_2Cl_2) using a gradient of 0 – 50% acetone in CH_2Cl_2 to give 165 mg (67%) of **7** as a yellow solid. Yield range (2 runs): 66-67%; average: 66%.

^{1}H NMR (500 MHz, CDCl$_3$): δ 8.82 (s, 1H), 8.09 (s, 1H), 7.56 – 7.51 (m, 2H), 7.45 – 7.38 (m, 4H), 7.32 – 7.25 (m, 2H), 7.23 – 7.16 (m, 1H), 7.09 (d, *J* = 4.6 Hz, 1H), 6.91 (d, *J* = 4.6 Hz, 1H), 6.87 – 6.80 (m, 4H), 5.22 (d, *J* = 7.2 Hz, 1H), 4.83 (d, *J* = 7.2 Hz, 1H),), 4.66 (d, *J* = 7.2 Hz, 1H), 4.62 (td, *J* = 7.6, 2.5 Hz, 1H), 4.53 – 4.46 (m, 1H), 3.98 (d, *J* = 3.2 Hz, 1H), 3.87 – 3.79 (m, 1H), 3.78 (s, 6H), 3.60 – 3.51 (m, 1H), 3.48 (dd, *J* = 10.5, 4.0 Hz, 1H), 3.40 (dd, *J* = 10.5, 4.0 Hz, 1H), 3.24 (s, 3H), 3.20 (s, 3H), 0.87 (t, *J* = 9.2 Hz, 2H), -0.02 (s, 9H).

^{13}C NMR (126 MHz, $CDCl_3$): δ 160.9, 158.5, 157.6, 147.4, 144.9, 136.2 (2C), 130.2 (2C), 130.2 (2C), 129.2, 128.4 (2C), 127.9 (2C), 126.8, 124.1, 123.1, 117.1, 114.1, 113.2 (4C), 102.4, 95.2, 86.3, 84.9, 82.5, 77.6, 75.3, 66.4, 62.7, 55.3 (2C), 41.5, 35.3, 18.2, -0.7 (3C, $-Si(CH_3)_3$).

HRMS: Calculated for $C_{42}H_{51}N_6O_7Si$ $[M+H]^+$: 779.3588; Found: 779.3578.

(2R,3R,4R,5R)-2-((bis(4-methoxyphenyl)(phenyl)methoxy)methyl)-5-cyano-5-(4-(((E)-(dimethylamino)methylene)amino)pyrrolo[2,1-f][1,2,4]triazin-7-yl)-4-((2-(trimethylsilyl)ethoxy)-methoxy)tetrahydrofuran-3-yl (2-cyanoethyl) diisopropylphosphoramidite (1):

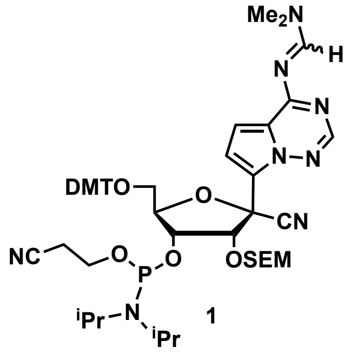

1

To a flame-dried flask under Ar was added **7** (61.1 mg, 0.0771 mmol), DIPEA (71.1 μL, 0.423 mmol) and 1,2-DCE (1 mL). The reaction was stirred at room temperature for 10 minutes. Next, 2-cyanoethyldiisopropylchloro-phosphorodiamidite (25.1 μL, 0.115 mmol) was added dropwise and stirred for 2 hours at room temperature. The reaction mixture was concentrated *in vacuo* and purified by chromatography on neutralized SiO_2 (neutralized by washing with 2 column volumes of 10% Et_3N in CH_2Cl_2) using a gradient from 0 **–** 50% EtOAc in CH_2Cl_2 to give 50 mg (66%) of **1** as a white solid. Yield range (2 runs): 54-66%; average: 60%.

^{1}H NMR (500 MHz, $CDCl_3$): δ 8.89 – 8.71 (m, 1H), 8.14 – 8.08 (m, 1H), 7.57 – 7.49 (m, 2H), 7.46 – 7.37 (m, 4H), 7.33 – 7.25 (m, 2H), 7.24 – 7.16 (m, 1H), 7.03 (d, *J* = 4.5 Hz, 0.6H), 6.98 (d, *J* = 4.5 Hz, 0.4H), 6.90 (dd, *J* = 4.6, 1.3 Hz, 1H), 6.87 – 6.80 (m, 4H), 5.46 (d, *J* = 4.6 Hz, 0.6H), 5.43 (d, *J* = 4.6 Hz, 0.4H), 4.85 (dd, *J* = 9.8, 6.2 Hz, 0.8H), 4.78 (dd, *J* = 9.8, 6.2 Hz, 1.2H), 4.72 (dt, *J* = 10.2, 5.7 Hz, 0.6H), 4.59 (dt, *J* = 10.2, 4.4 Hz, 0.4H), 4.49 (app q, *J* = 4.8 Hz, 0.6H), 4.46 (app q, *J* = 4.8 Hz, 0.4H), 3.82 – 3.75 (m, 6H), 3.69 – 3.27 (m, 8H), 3.24 (s, 3H), 3.21 (s, 3H), 2.43 (t, *J* = 6.6 Hz, 1H), 2.36 – 2.21 (m, 1H), 1.09 (d, *J* = 6.8 Hz, 3.5H), 1.05 (d, *J* = 6.8 Hz, 2.5H), 0.99 (d, *J* = 6.7 Hz, 3.5H), 0.94 (d, *J* = 6.7 Hz, 2.5H), 0.92 – 0.55 (m, 2H), -0.02 (s, 3.5H), -0.04 (s, 5.5H). (60:40 ratio of phosphorous diastereomers in based on ^{31}P NMR).

^{31}P NMR (202 MHz, $CDCl_3$): δ 150.13, 149.71.

^{13}C NMR (126 MHz, $CDCl_3$) (major phosphorous diastereomer): δ 160.8, 158.5, 157.5, 147.4, 144.9, 136.2, 136.2, 130.4 (2C), 130.3 (2C), 128.6, 127.9 (2C), 126.8, 123.6, 123.3, 117.4, 116.7, 113.3, 113.2 (4C), 102.5, 94.7, 86.3, 84.9, 83.8, 82.1, 82.0, 78.8, 77.8, 66.0, 62.7, 58.5, 58.1, 55.3 (2C), 43.3, 41.5, 35.3, 24.6, 24.5, 24.4, 20.0, 19.9, 17.86, -1.2 (3C, $-Si(CH_3)_3$)).

(**minor phosphorous diastereomer**): δ 160.7, 158.5, 157.6, 147.4, 144.9, 136.2, 136.1, 130.3 (2C), 130.3 (2C), 128.4, 127.9 (2C), 126.8, 123.5, 123.4, 117.5, 116.4, 113.2, 112.8 (4C), 102.6, 93.5, 86.3, 84.9, 83.2, 82.1, 81.5, 79.4, 77.9, 65.9, 62.9, 58.4, 58.3, 55.3 (2C), 43.2, 41.5, 35.3, 24.6, 24.6, 24.4, 20.1, 19.9, 18.0, -0.3 (3C, $-Si(CH_3)_3$).

HRMS: Calculated for $C_{51}H_{68}N_8O_8PSi$ $[M+H]^+$: 979.4667; Found: 979.4622.

III. Protecting Group Studies Towards the Synthesis of the GS-441524 Phosphoramidite.

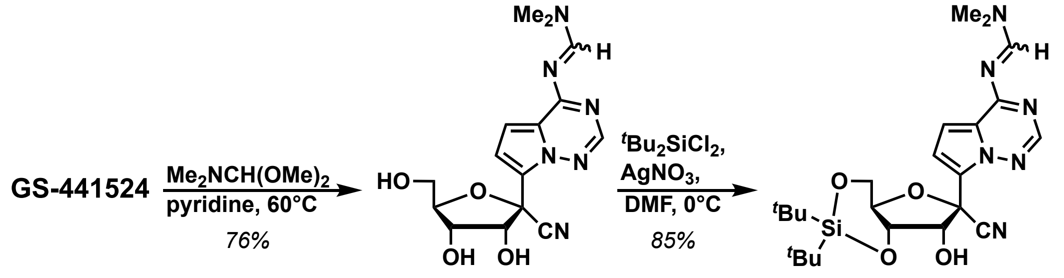

4 **3b**

Scheme 1. Synthesis of **3b**.

N'-(7-((2R,3R,4S,5R)-2-cyano-3,4-dihydroxy-5-(hydroxymethyl)tetrahydrofuran-2-yl)pyrrolo[2,1-f][1,2,4]triazin-4-yl)-N,N-dimethylformimidamide (4).

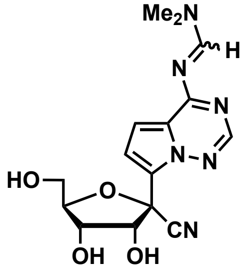

4

To a flame-dried flask under Ar was added **GS-441524** (100 mg, 0.343 mmol), *N,N*-dimethylformamide dimethyl acetal (0.171 g, 1.44 mmol), and pyridine (1 mL). The reaction mixture was stirred at room temperature for 24 hours. The reaction mixture was then concentrated *in vacuo* to remove pyridine and was diluted with EtOAc (80 mL). The EtOAc layer was subsequently washed with brine (3 X 50 mL), dried over anhydrous Na_2SO_4 and concentrated *in vacuo*. The crude product was purified by SiO_2 chromatography (gradient 0 – 20% CH_3OH in $CHCl_3$) to give 100 mg (85%) of **4** as an off-white solid. Yield range (2 runs): 68-85%; average: 76%.

^{1}H NMR (500 MHz, DMSO-*d*$_6$): δ 9.45 (s, 1H), 8.66 (s, 1H), 7.51 (d, *J* = 4.5 Hz, 1H), 7.35 (d, *J* = 4.5 Hz, 1H), 6.64 (d, *J* = 6.3 Hz, 1H), 5.74 (d, *J* = 5.1 Hz, 1H), 5.44 (app t, *J* = 5.7 Hz, 1H), 5.21 (app t, *J* = 5.8 Hz, 1H), 4.61 (app q, *J* = 4.5 Hz, 1H), 4.52 (app q, *J* = 5.2 Hz, 1H), 4.22 – 4.14 (m, 1H), 4.09 – 4.01 (m, 1H), 3.75 (s, 3H), 3.69 (s, 3H).

^{13}C NMR (126 MHz, DMSO-*d*$_6$): δ 160.2, 158.1, 147.3, 123.8, 122.6, 117.3, 111.9, 101.8, 85.5, 78.4, 74.2, 70.1, 60.9, 41.1, 34.9.

HRMS: Calculated for $C_{15}H_{19}N_6O_4$ $[M+H]^+$: 347.1468; Found: 347.1455.

N'-(7-((4aR,6R,7R,7aS)-2,2-di-tert-butyl-6-cyano-7-hydroxytetrahydro-4H-furo[3,2-d][1,3,2]dioxasilin-6-yl)pyrrolo[2,1-f][1,2,4]triazin-4-yl)-N,N-dimethylformimidamide (3b):

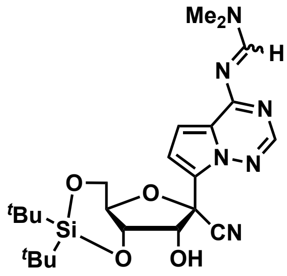

3b

To a flame-dried flask under Ar was added **4** (100 mg, 0.288 mmol), di-*tert*-butyl-dichlorosilane (91.1 mL, 0.433 mmol), $AgNO_3$ (73.5 mg, 0.433 mmol), and DMF (2 mL). The reaction mixture was stirred at 0 ºC for 90 minutes. The reaction was quenched with water (5 mL) and diluted with EtOAc (40 mL). The EtOAc layer was subsequently washed with brine (3 X 40 mL), dried over anhydrous Na_2SO_4 and concentrated *in vacuo*. The crude product was purified by SiO_2 chromatography

(gradient 0 – 100% EtOAc in CH_2Cl_2) to give 120 mg (85%) of **3b** as an off-white solid. Yield range (2 runs): 85-85%; average: 85%.

^1H and ^{13}C NMR are shown in **Section II.**

N'-(7-((4aR,6R,7R,7aR)-2,2-di-tert-butyl-7-((tert-butyldimethylsilyl)oxy)-6-cyanotetrahydro-4H-furo[3,2-d][1,3,2]dioxasilin-6-yl)pyrrolo[2,1-f][1,2,4]triazin-4-yl)-N,N-dimethylformimidamide (5a:

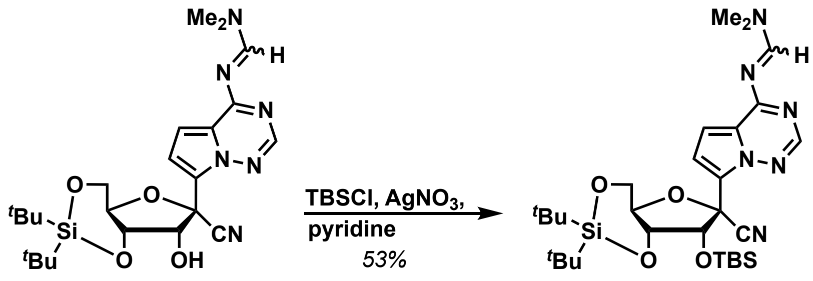

3a 5a

To a flame-dried flask under Ar was added **3a** (40.5 mg, 0.0822 mmol), *t*-butyldimethylsilylchloride (17.5 mg, 0.115 mmol), $AgNO_3$ (19.5 mg, 0.115 mmol), and pyridine (1 mL). The reaction mixture was stirred at room temperature for 48 hours under dark conditions; the reaction flask was wrapped in aluminum foil. The reaction mixture was concentrated *in vacuo* to remove pyridine, followed by the dilution in CH_2Cl_2 (40 mL). The CH_2Cl_2 layer was subsequently washed with saturated $NaHCO_3$ (3 X 30 mL), dried over anhydrous Na_2SO_4, and concentrated *in* vacuo. The crude product was purified by SiO_2 chromatography (gradient of 0 **–** 50% EtOAc in CH_2Cl_2 to give 26 mg (53%) of **5a** as a yellow solid. Yield range (2 runs): 50-53%; average: 52%.

^{1}H NMR (500 MHz, CDCl$_3$): δ 8.84 (s, 1H), 8.03 (s, 1H), 7.04 (d, *J* = 4.6 Hz, 1H), 6.89 (d, *J* = 4.6 Hz, 1H), 5.38 (d, *J* = 8.7 Hz, 1H), 4.66 (td, *J* = 10.4, 5.1 Hz, 1H), 4.39 – 4.32 (m, 2H), 4.05 (dd, *J* = 10.7, 9.1 Hz, 1H), 3.25 (s, 3H), 3.22 (s, 3H), 1.09 (s, 9H), 1.06 (s, 9H), 0.81 (s, 9H), -0.16 (s, 3H), -0.23 (s, 3H).

^{13}C NMR (126 MHz, CDCl$_3$): δ 161.0, 157.8, 147.4, 124.0, 123.2, 117.0, 114.6, 102.4, 80.7, 78.8, 77.0, 75.6, 68.0, 41.6, 35.4, 27.4 (3C, ^{t}Butyl), 27.2 (3C, ^{t}Butyl), 25.5 (3C, ^{t}Butyl), 22.7, 20.3, 18.0, -5.0, -5.2.

HRMS: Calculated for $C_{29}H_{49}N_6O_4Si_2$ $[M+H]^+$: 601.3354; Found: 601.3318

IV. Preparation of GS-441524-Containing RNA.

RNA was synthesized using standard solid-phase synthesis on a BioAutomation MerMade4 DNA/RNA synthesizer. Remdesivir-containing phosphoramidite **1** was synthesized as described in **Section II**. All other phosphoramidites and reagents were purchased from Glen Research Corporation. The CPG universal solid support (1.0 µmol scale; MM1-3500) was purchased from BioAutomation Technologies. Phosphoramidites and reagents were freshly prepared prior to synthesis. Trap packs (NC1758846) were added to the anhydrous acetonitrile (40-4050-50, Glen Research) and activator (30-3140-57, Glen Research) solutions. Automated RNA synthesis was paused immediately prior to the incorporation of **1**. Freshly prepared **1** (30.0 mg) was dissolved in anhydrous acetonitrile (200.0 µL, ~50 mM) and was loaded onto the instrument prior to incorporation for on-system coupling. Reaction conditions for the synthesis of

RNA-1 were optimized based on vendor recommendations and literature precedence (**Supplemental Table 1**).[1-5]

Supplemental Table 1. Reagent table of optimized coupling conditions used for RNA synthesis.

Composition of Reagent	Reaction Time on MerMade4	Number of times reagent added during one base coupling	Product number from Glen Research
Deblocking Mix: 3 % dichloroacetic acid in dichloromethane	1-minute rxn	2X	40-4040-57
Cap A: Tetrahydrofuran:acetic anhydride:pyridine (80:10:10)	1-minute rxn	2X	40-4110-57
Cap B: 16 % 1-methylimidazole in tetrahydrofuran	1-minute rxn	2X	40-4220-57
Oxidation Solution: 0.02 M I_2 in tetrahydrofuran:pyridine:water (88:10:2)	1-minute rxn	2X	40-4032-57
Activator Solution: 0.25 M 5-ethylthio-1H-tetrazole in anhydrous acetonitrile	12-minute rxn	2X [a] or 3X[b]	30-3140-57

[a] Standard RNA phosphoramidites were coupled onto the resin 2X. [b] RNA phosphoramidite **1** was coupled onto the resin 3X.

Following the synthesis of **RNA-1** (5'–CCGGGCGGCR–3', where R = **1**), the resin was dried under N_2 for 1 hour before being transferred to a fritted reaction vessel. Concentrated aqueous NH_4OH (2.5 mL) was added, and the aluminum foil-covered vessel was placed in a shaker for 30-hours at room temperature. After the removal of the resin, the solution was filtered into a centrifuge tube and diluted with DNase/RNase-free water (2 mL, cat# 10977015 Thermo Fisher). The solution was transferred into microcentrifuge tubes and concentrated *in vacuo* in a SpeedVac. The crude sample was completely dry before being deprotected with 1.0 M tetrabutylammonium fluoride (TBAF) in tetrahydrofuran (2 mL) for 12-hours in a 4-mL glass vial equipped with a stir bar. The solution was concentrated *in vacuo* and the crude material was purified by HPLC. **RNA-1** was HPLC purified on an Agilent 1200 series instrument equipped with a diode array detector and a PLRP-S column (8 µmol, 100 Å, 4.6 X 150 mm, Agilent Technologies). The analysis method (2.750 mL/min flow rate) involved isocratic 100 mM triethylammonium acetate (TEAA, pH 7.0, Sigma Aldrich; 0-5 minutes, Solvent A) followed by a linear gradient to 10 % 100 mM TEAA:MeCN (1:1, 5-10 minutes, Solvent B) and finally a linear gradient of 10-70% 100 mM TEAA/MeCN (1:1, 10-45 minutes, Solvent B). Wavelengths monitored = 260 nm. After purification **RNA-1** was desalted with DNase/RNase-free water using an Illustra NAP-5 column (Sephadex G-25 DNA grade, GE Healthcare) according to the manufacturer's instructions. The desalted oligoribonucleotide was quantified by UV-vis Spectroscopy (using the molar extinction coefficient of 765 M^{-1} cm^{-1} for Remdesivir calculated at 260 nm in H_2O, **Supplemental Information V**) and confirmed by LC-MS. The purity was assessed by HPLC

reinjection of the purified oligonucleotides. LC-MS was performed on a Thermo Scientific LTQ XL instrument equipped with a diode array detector and using a Zorbax C-18 column (5 μm, 80 Å, 0.5 X 150 mm, Agilent Technologies). The analysis method (15 μL/min flow rate) involved 15 mM aqueous NH_4OAc containing 2% CH_3CN followed by a linear gradient of 2-15% CH_3CN (0-15 min) and 25-60% CH_3CN (15-25 min). Wavelengths monitored = 260 nm.

V. Molar Extinction Coefficient Calculation of Remdesivir.

The extinction coefficient of Remdesivir was calculated as follows: a glass vial was dried *in vacuo* for 48 hours before addition of a known mass of commercial Remdesivir. The charged vial with dried *in vacuo* for an additional 24 hours before dissolving the Remdesivir in H_2O such that the exact concentration of the solution was known. Beer's Law plots were then constructed by taking the solution and diluting serially such that 6 samples of a known concentration could be measured at 260 nm using a UV spectrometer (all absorbances < 1.0) using quartz cuvettes (Starna Cells, Inc., 10 mm path length). The slope of the line absorbance vs concentration (in molar, M) is the extinction coefficient ε_{260} in $M^{-1}cm^{-1}$. This process was repeated four times with four different stock solutions, the slopes averaged, and standard deviation calculated using Microsoft Excel. The ε_{260} calculated for Remdesivir was 765 ± 77 M^{-1} cm^{-1} using the nearest-neighbors model equation (**Equation 1**).

$$\varepsilon_{260} = \sum_{1}^{N-1} \varepsilon \text{ Nearest Neighbor} - \sum_{1}^{N-1} \varepsilon \text{ Individual Bases}$$

VI. NMR Spectroscopic Data.

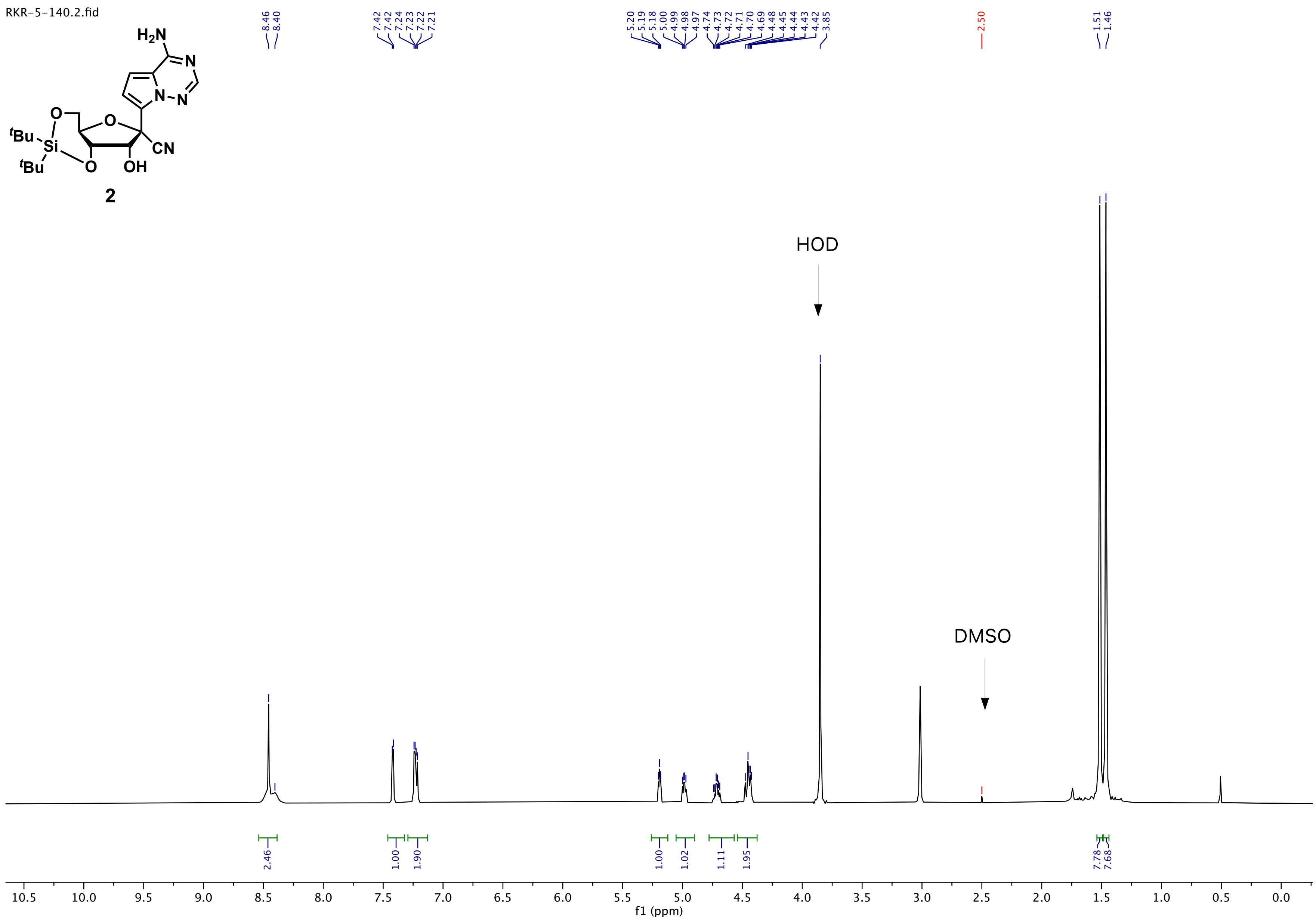

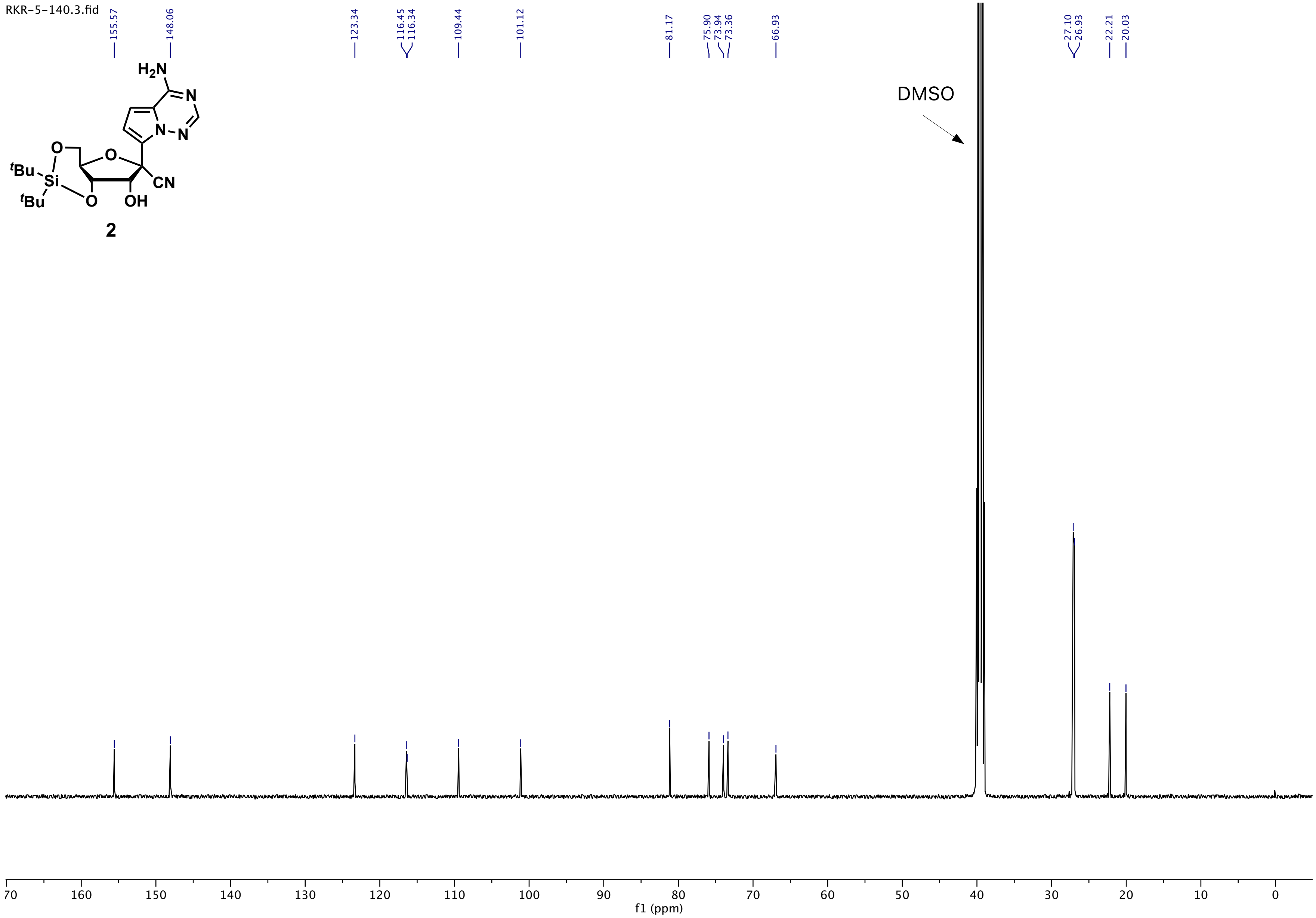
RKR-5-140.3.fid
155.57
148.06
123.34
116.45
116.34
109.44
101.12
81.17
75.90
73.94
73.36
66.93
27.10
26.93
22.21
20.03
H2N
N
N
N
O
O
O
tBu
Si
tBu
OH
CN
2
DMSO
170 160 150 140 130 120 110 100 90 80 70 60 50 40 30 20 10 0
f1 (ppm)

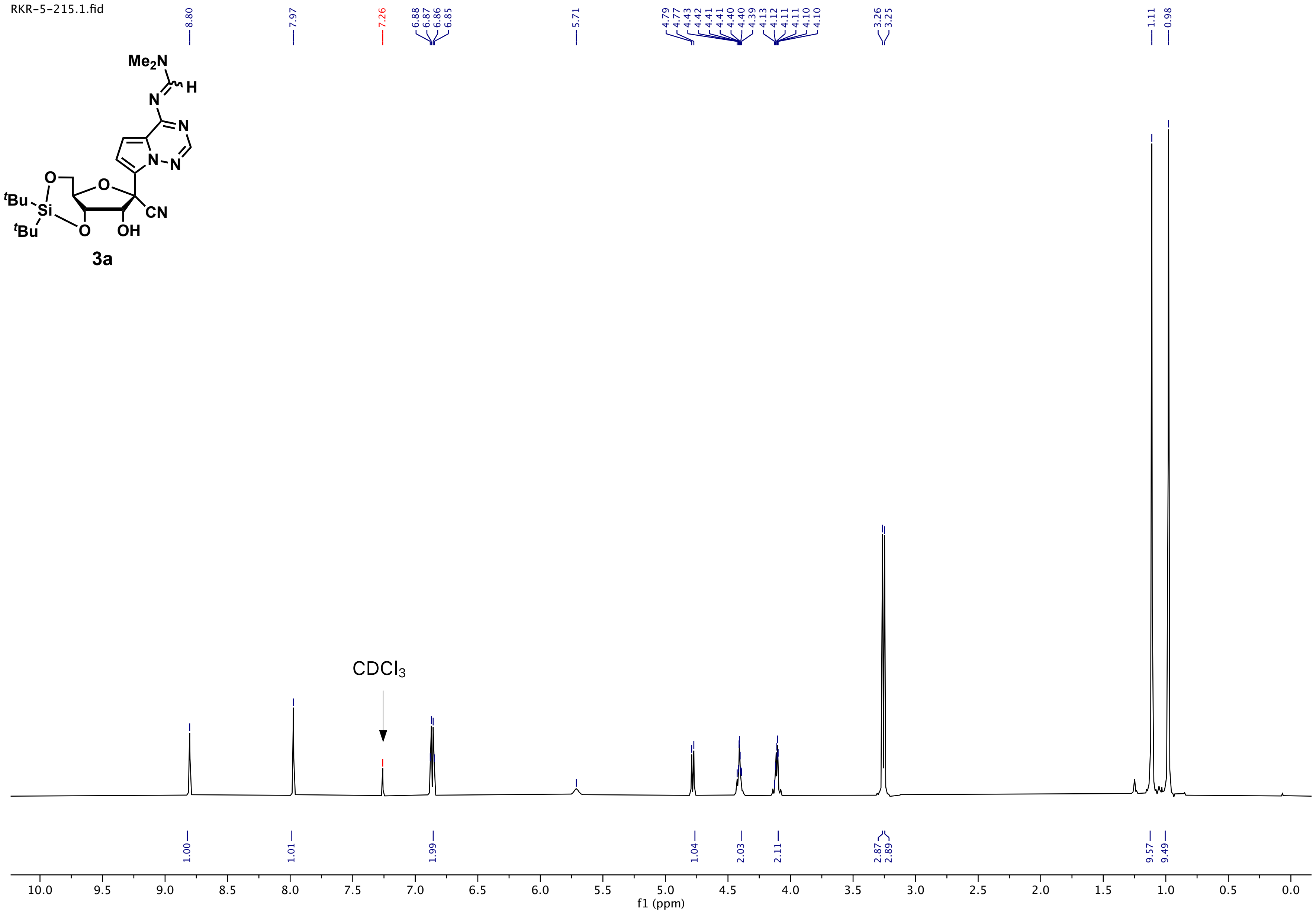

RKR-5-215.1.fid
8.80
7.97
7.26
6.88
6.87
6.86
6.85
5.71
4.79
4.77
4.43
4.42
4.41
4.41
4.40
4.40
4.39
4.13
4.12
4.11
4.11
4.10
4.10
3.26
3.25
1.11
0.98
Me2N
H
N
N
N
N
O
O
tBu
Si
tBu
O
OH
CN
3a
CDCl3
1.00
1.01
1.99
1.04
2.03
2.11
2.87
2.89
9.57
9.49
f1 (ppm)

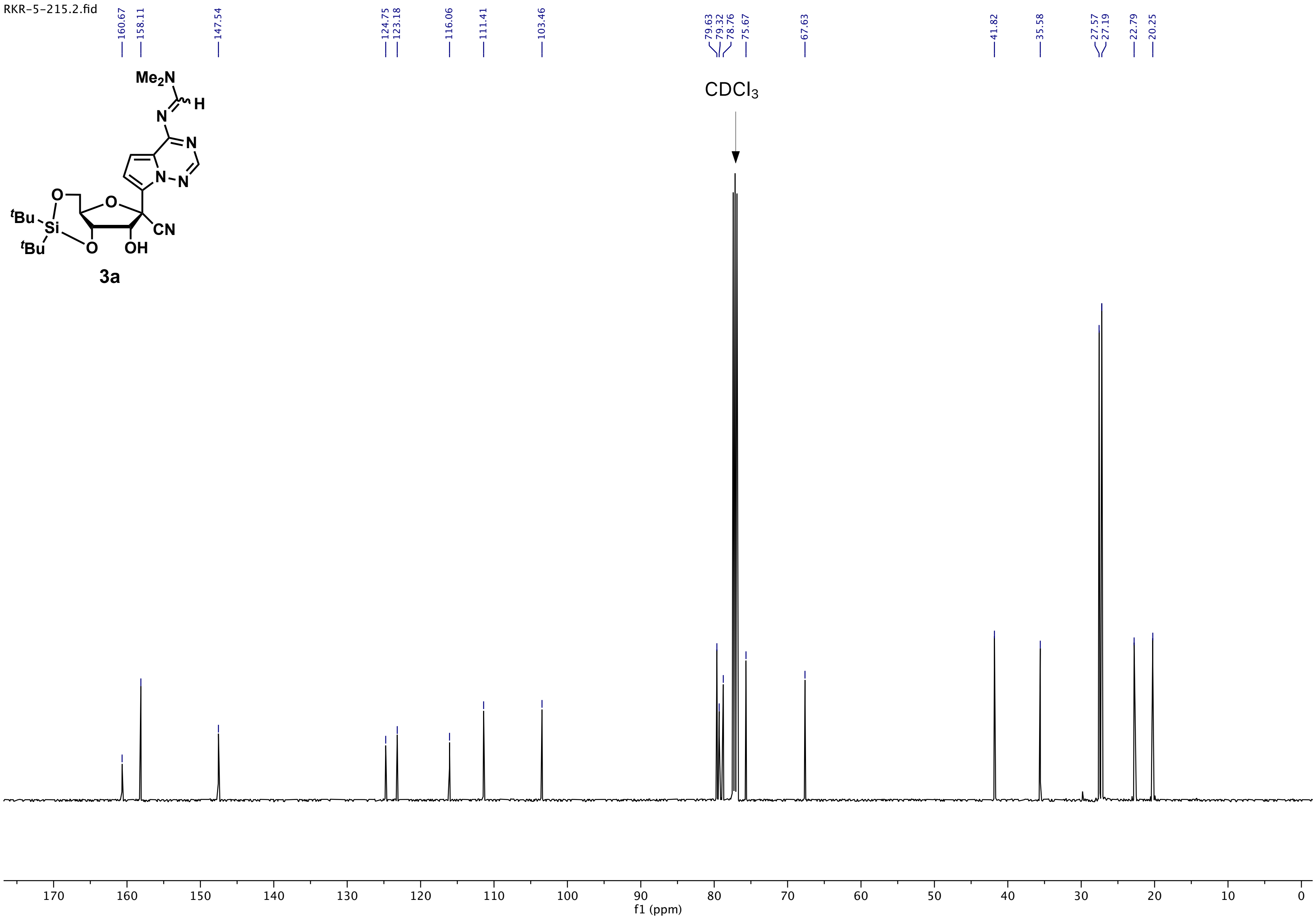
RKR-5-215.2.fid
160.67
158.11
147.54
124.75
123.18
116.06
111.41
103.46
79.63
79.32
78.76
75.67
67.63
41.82
35.58
27.57
27.19
22.79
20.25
CDCl3
Me2N
H
N
N
N
N
O
O
tBu
Si
tBu
O
O
OH
CN
3a
170
160
150
140
130
120
110
100
90
80
70
60
50
40
30
20
10
0
f1 (ppm)

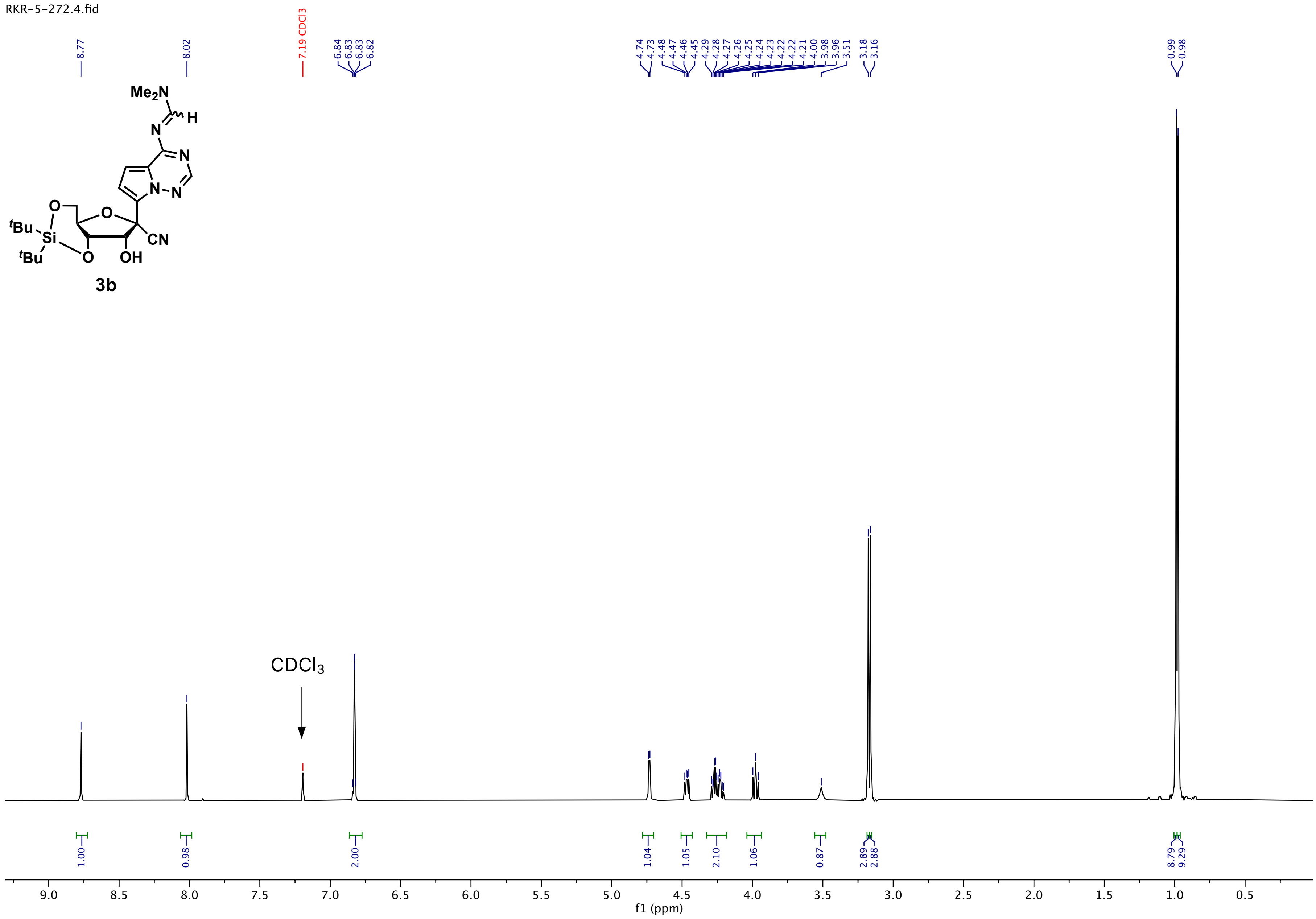
RKR-5-272.4.fid
8.77
8.02
7.19 CDCl3
6.84 6.83 6.83 6.82
4.74 4.73 4.48 4.47 4.46 4.45 4.29 4.28 4.27 4.26 4.25 4.24 4.23 4.22 4.22 4.21 4.00 3.98 3.96 3.51
3.18 3.16
0.99 0.98
Me2N
H
N
N
N
N
O
O
O
tBu
Si
tBu
OH
CN
3b
CDCl3
1.00
0.98
2.00
1.04
1.05
2.10
1.06
0.87
2.89 2.88
8.79 9.29
9.0 8.5 8.0 7.5 7.0 6.5 6.0 5.5 5.0 4.5 4.0 3.5 3.0 2.5 2.0 1.5 1.0 0.5
f1 (ppm)

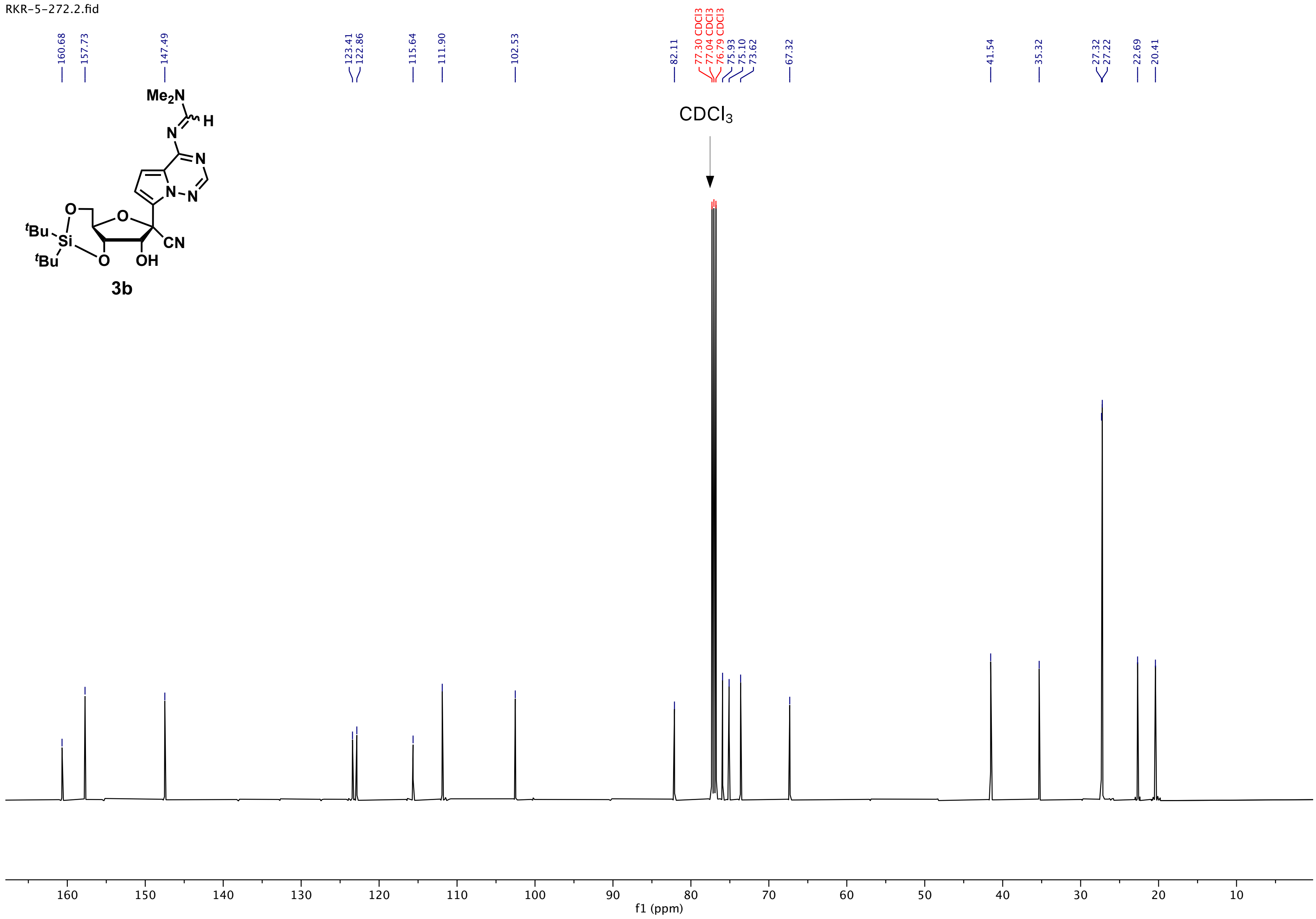
RKR-5-272.2.fid
160.68
157.73
147.49
123.41
122.86
115.64
111.90
102.53
82.11
77.30 CDCl3
77.04 CDCl3
76.79 CDCl3
75.93
75.10
73.62
67.32
41.54
35.32
27.32
27.22
22.69
20.41
CDCl$_3$
Me$_2$N
H
N
N
N
N
O
O
O
^{t}Bu
Si
^{t}Bu
CN
OH
3b
160
150
140
130
120
110
100
90
80
70
60
50
40
30
20
10
f1 (ppm)

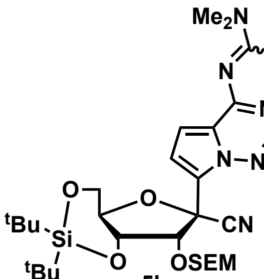
Me2N
H
N
N
N
N–N
O
O
tBu
Si
tBu
O
CN
OSEM

5b

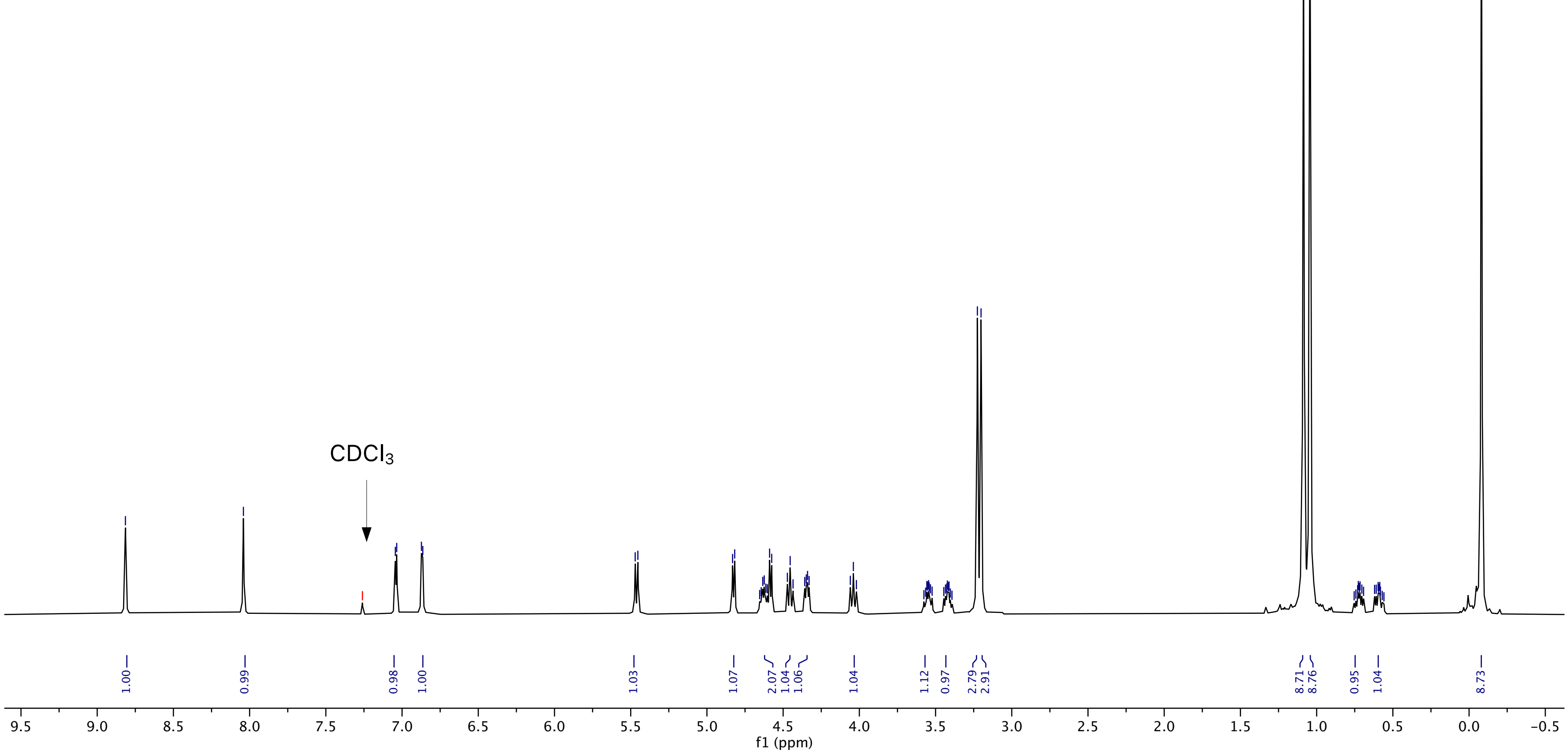
RKR-5-203.1.fid
8.81
8.04
7.26
7.04 7.04 6.87 6.86
5.47 5.45 4.83 4.82 4.65 4.64 4.63 4.62 4.61 4.60 4.59 4.58 4.47 4.45 4.44 4.36 4.35 4.34 4.33 4.06 4.04 4.02 3.58 3.57 3.56 3.55 3.55 3.54 3.53 3.52 3.45 3.43 3.43 3.42 3.41 3.40 3.39 3.23 3.20
1.09 1.04 0.75 0.74 0.73 0.73 0.72 0.72 0.70 0.69 0.62 0.61 0.60 0.59 0.59 0.58 0.57 0.56 -0.08
CDCl3
1.00 0.99 0.98 1.00 1.03 1.07 2.07 1.04 1.06 1.04 1.12 0.97 2.79 2.91 8.71 8.76 0.95 1.04 8.73
9.5 9.0 8.5 8.0 7.5 7.0 6.5 6.0 5.5 5.0 4.5 4.0 3.5 3.0 2.5 2.0 1.5 1.0 0.5 0.0 –0.5
f1 (ppm)

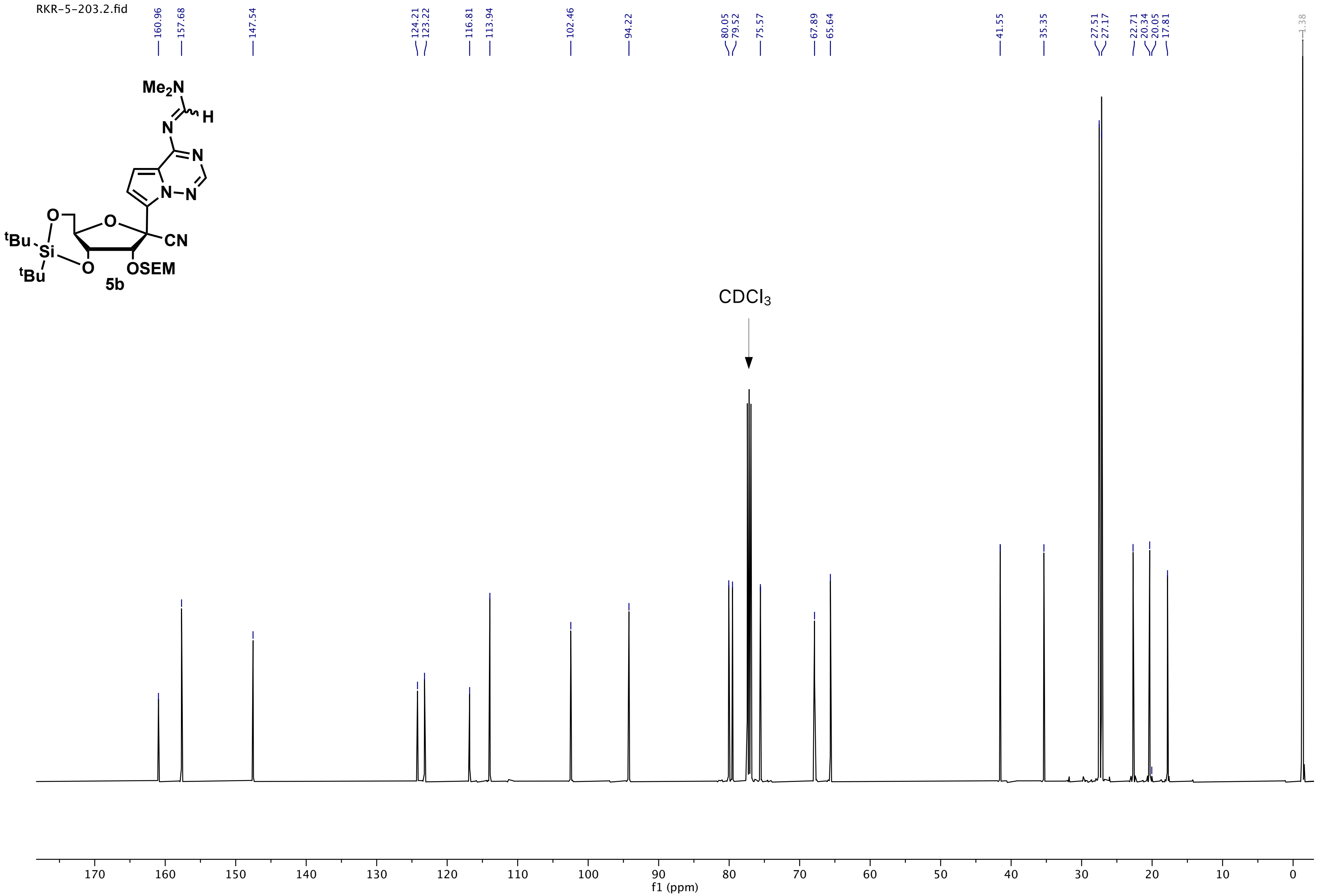
RKR-5-203.2.fid
160.96
157.68
147.54
124.21
123.22
116.81
113.94
102.46
94.22
80.05
79.52
75.57
67.89
65.64
41.55
35.35
27.51
27.17
22.71
20.34
20.05
17.81
1.38
Me2N
H
N
N
N
N
O
O
tBu
Si
tBu
O
CN
OSEM
5b
CDCl3
170 160 150 140 130 120 110 100 90 80 70 60 50 40 30 20 10 0
f1 (ppm)

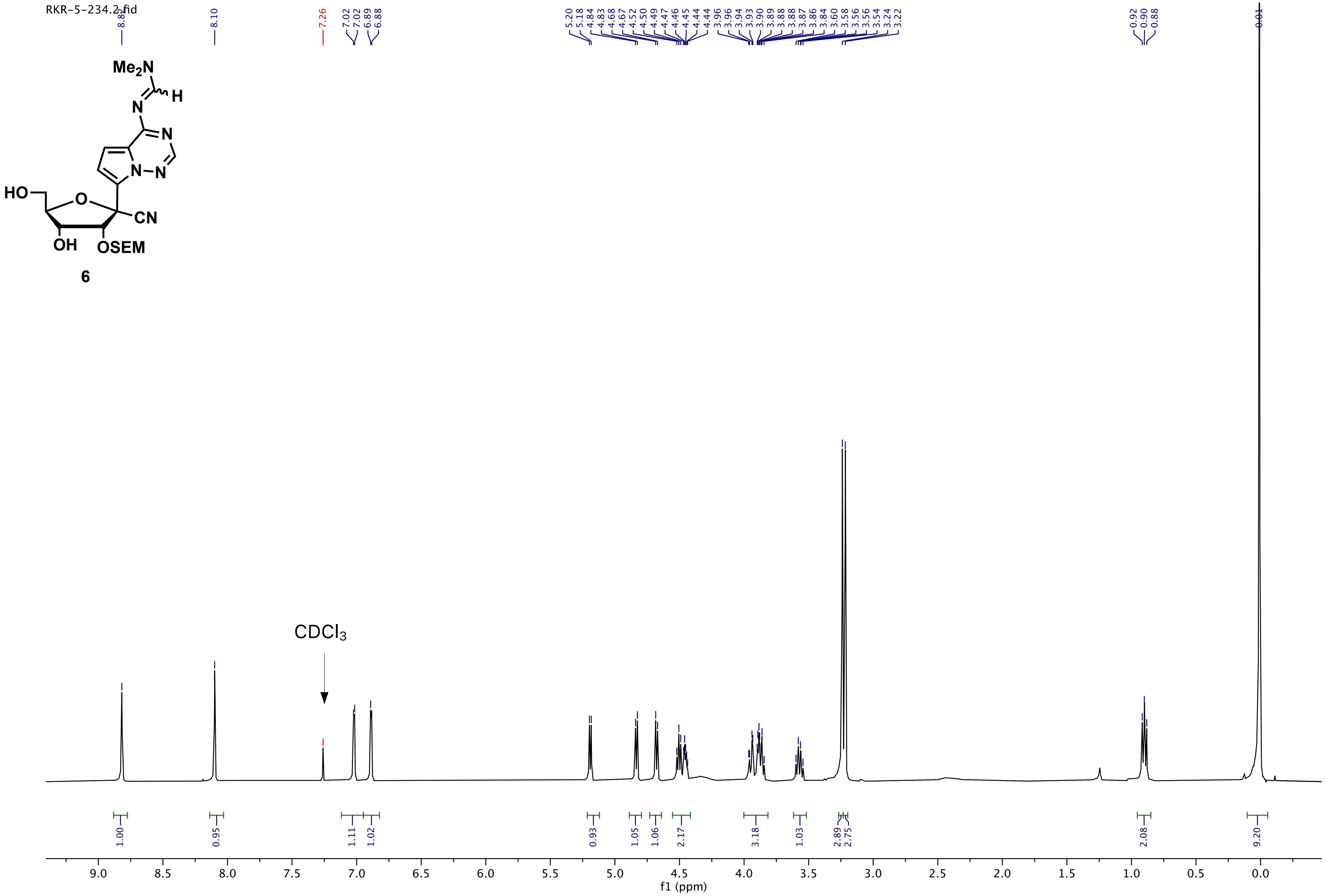

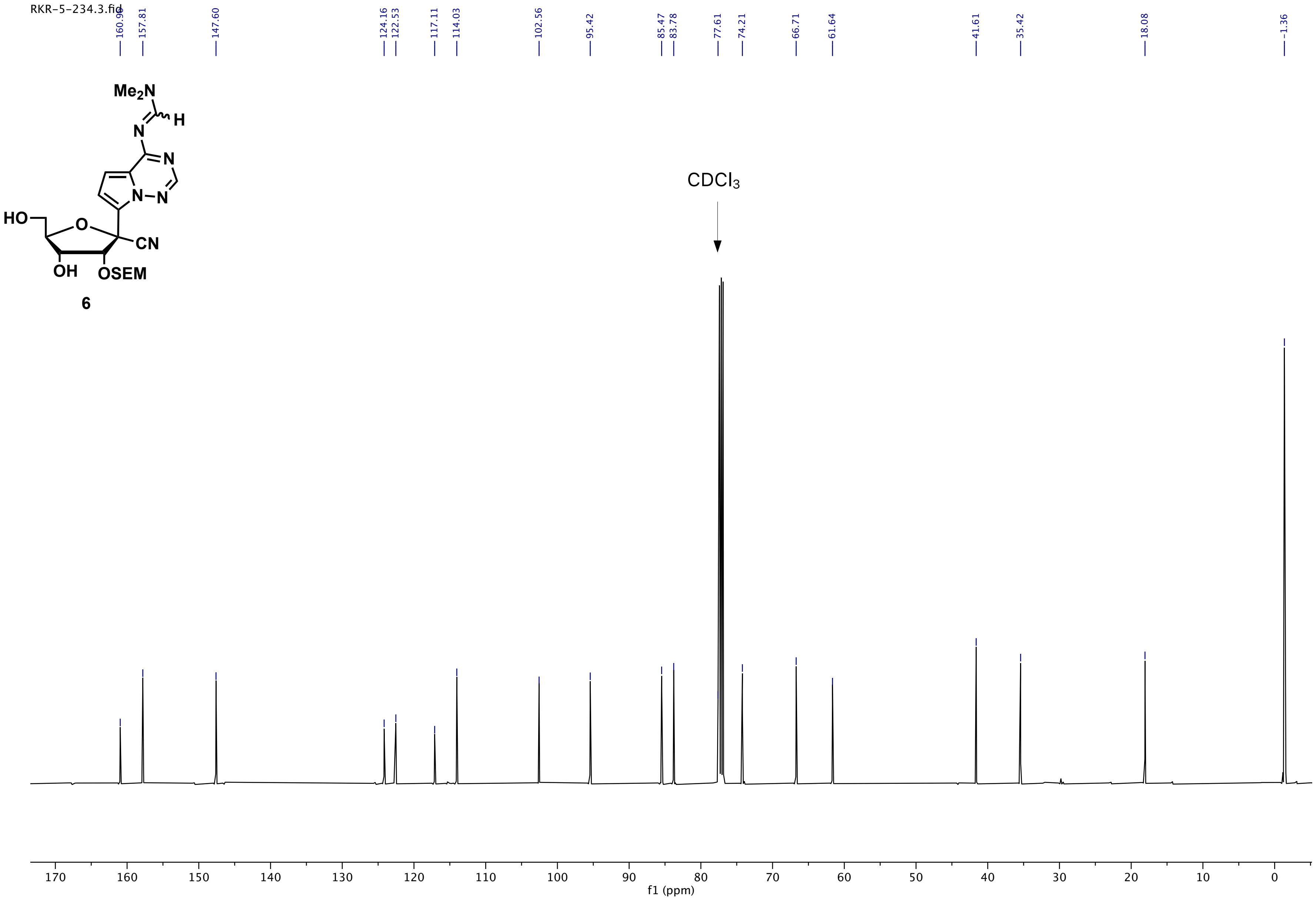
RKR-5-234.3.fid
160.96
157.81
147.60
124.16
122.53
117.11
114.03
102.56
95.42
85.47
83.78
77.61
74.21
66.71
61.64
41.61
35.42
18.08
-1.36
Me2N
H
N
N
N
N
HO
O
CN
OH
OSEM
6
CDCl3
170 160 150 140 130 120 110 100 90 80 70 60 50 40 30 20 10 0
f1 (ppm)

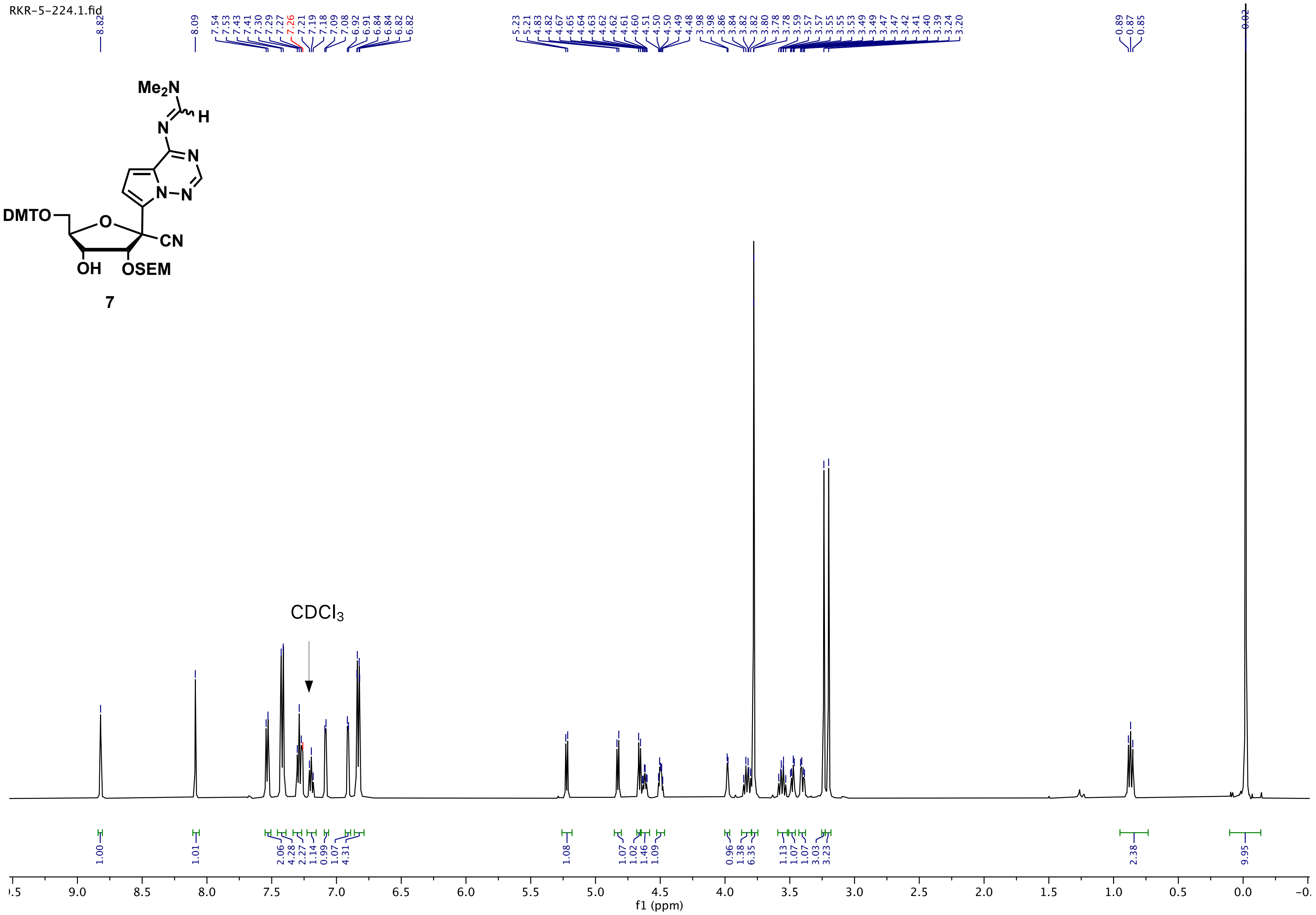
RKR-5-224.1.fid
8.82
8.09
7.54 7.53 7.43 7.41 7.30 7.29 7.27 7.26 7.21 7.19 7.18 7.09 7.08 6.92 6.91 6.84 6.84 6.82 6.82
5.23 5.21 4.83 4.82 4.67 4.65 4.64 4.63 4.62 4.62 4.61 4.60 4.51 4.50 4.50 4.49 4.48 3.98 3.98 3.86 3.84 3.82 3.82 3.80 3.78 3.78 3.59 3.57 3.57 3.55 3.55 3.53 3.49 3.49 3.47 3.47 3.42 3.41 3.40 3.39 3.24 3.20
0.89 0.87 0.85
0.02
Me2N
H
N
N
N
N
DMTO
O
CN
OH
OSEM
7
CDCl3
1.00 1.01 2.06 4.28 2.27 1.14 0.99 1.07 4.31 1.08 1.07 1.02 1.46 1.09 0.96 1.38 6.35 1.13 1.07 1.07 3.03 3.23 2.38 9.95
f1 (ppm)

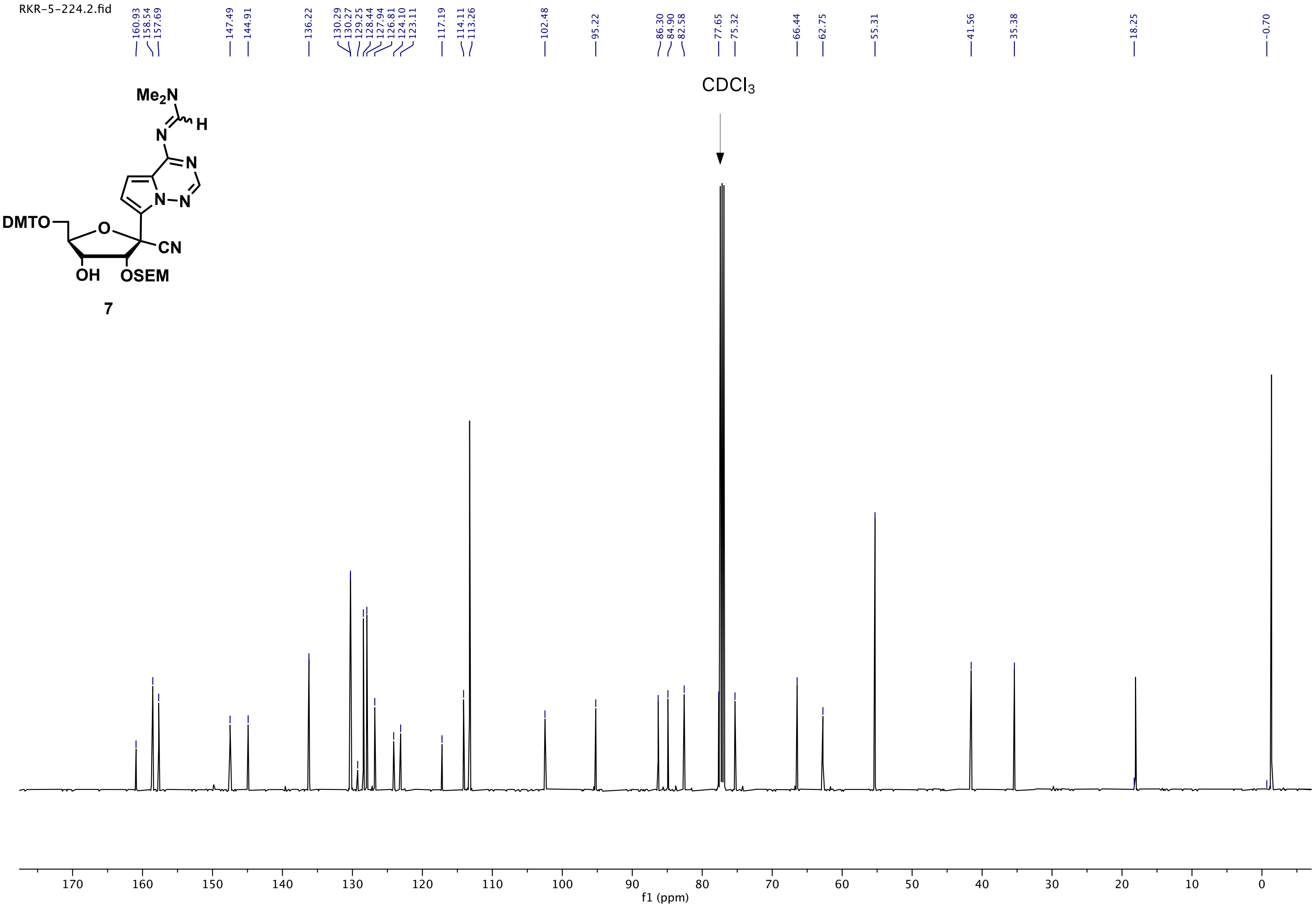
RKR-5-224.2.fid
160.93
158.54
157.69
147.49
144.91
136.22
130.29
130.27
129.25
128.44
127.94
126.81
124.10
123.11
117.19
114.11
113.26
102.48
95.22
86.30
84.90
82.58
77.65
75.32
66.44
62.75
55.31
41.56
35.38
18.25
-0.70
CDCl₃
Me₂N
N
H
N
N
N
N
DMTO
O
CN
OH
OSEM
7
170 160 150 140 130 120 110 100 90 80 70 60 50 40 30 20 10 0
f1 (ppm)

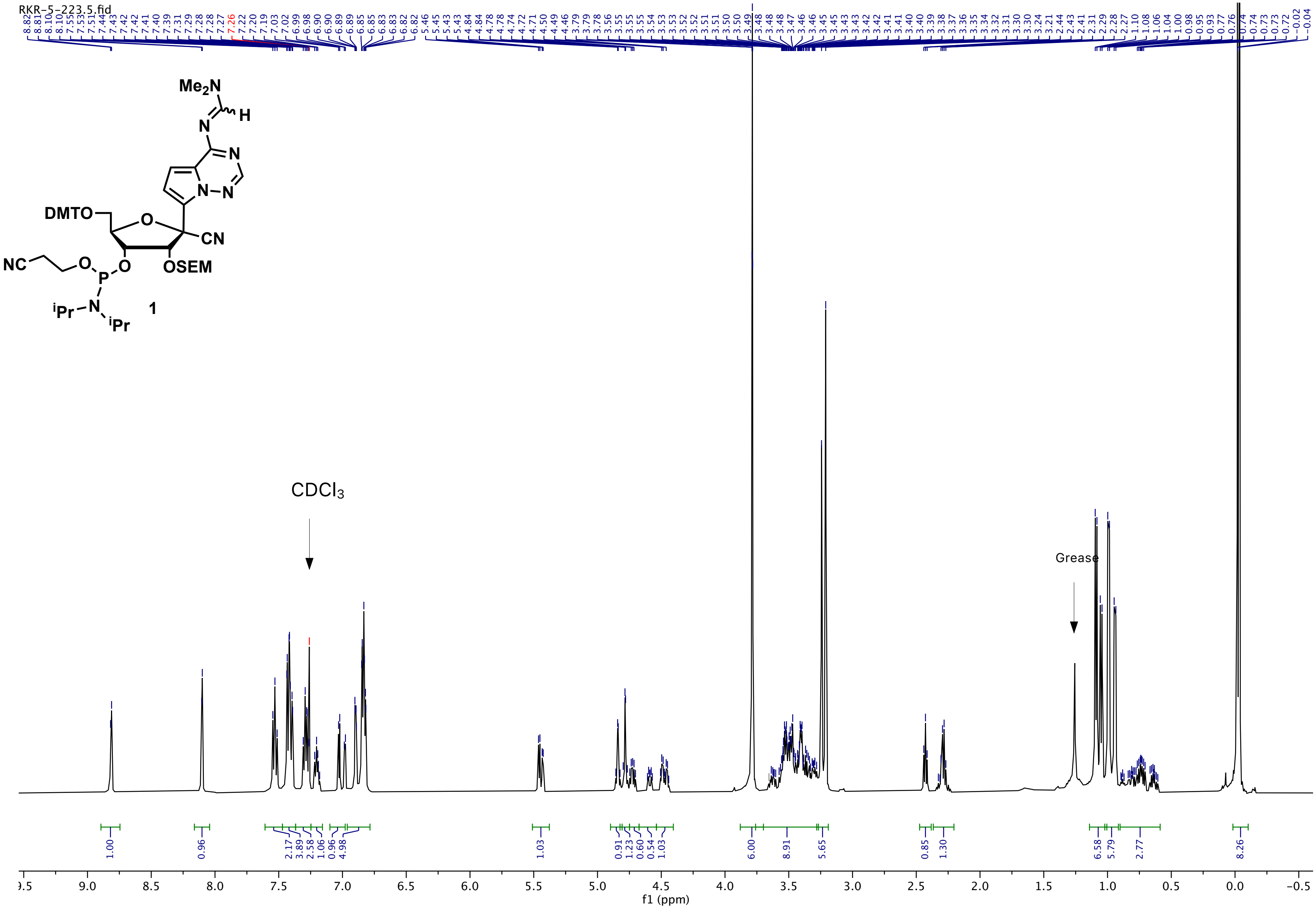
RKR-5-223.5.fid
Me2N
H
N
N
N
N
DMTO
O
CN
NC
O
P
O
OSEM
iPr
N
iPr
1
CDCl3
Grease
f1 (ppm)

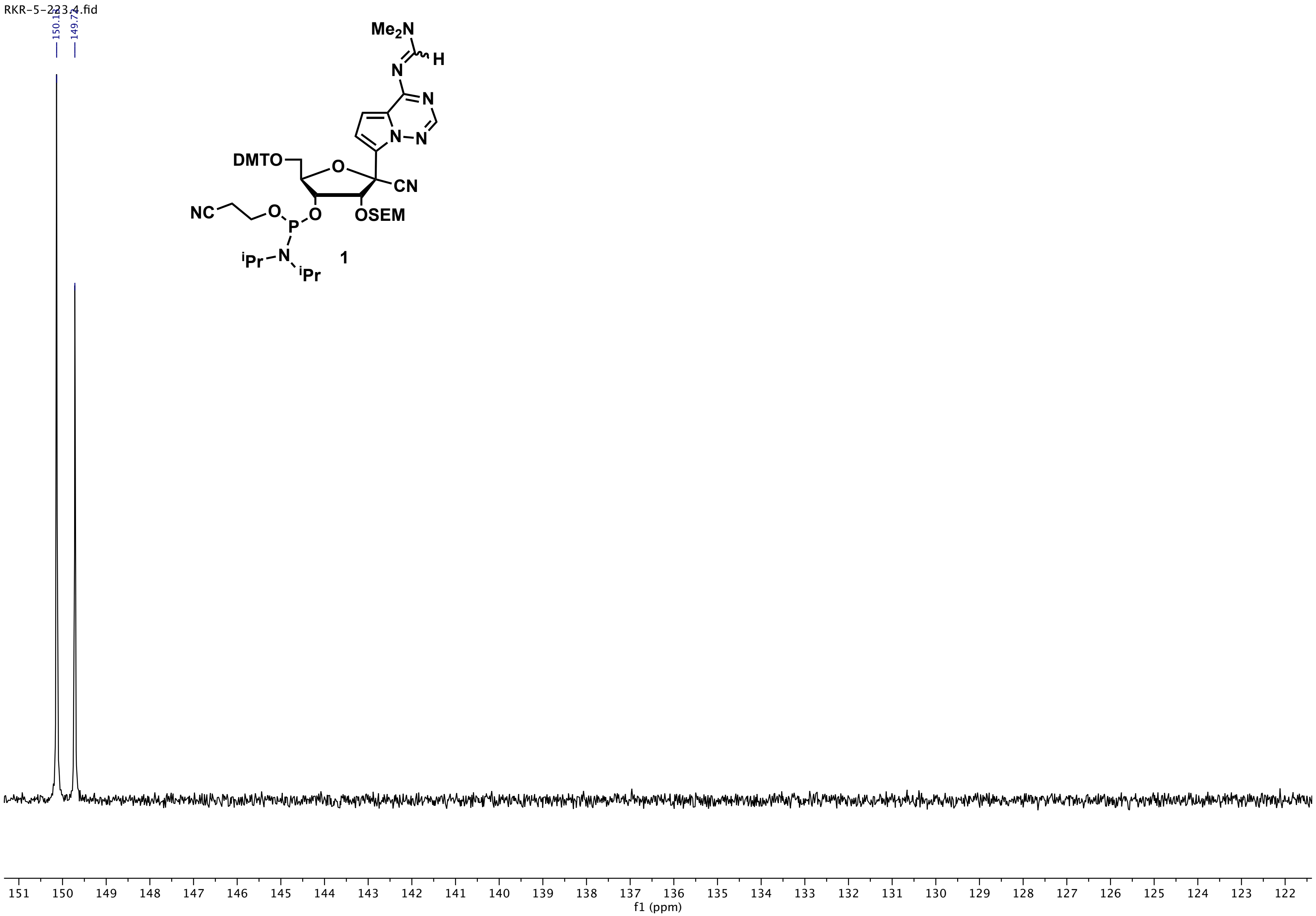
RKR-5-223.4.fid
150.1
149.7
Me2N
H
N
DMTO
O
CN
NC
O
P
OSEM
iPr
1
151
150
149
148
147
146
145
144
143
142
141
140
139
138
137
136
135
134
133
132
131
130
129
128
127
126
125
124
123
122
f1 (ppm)

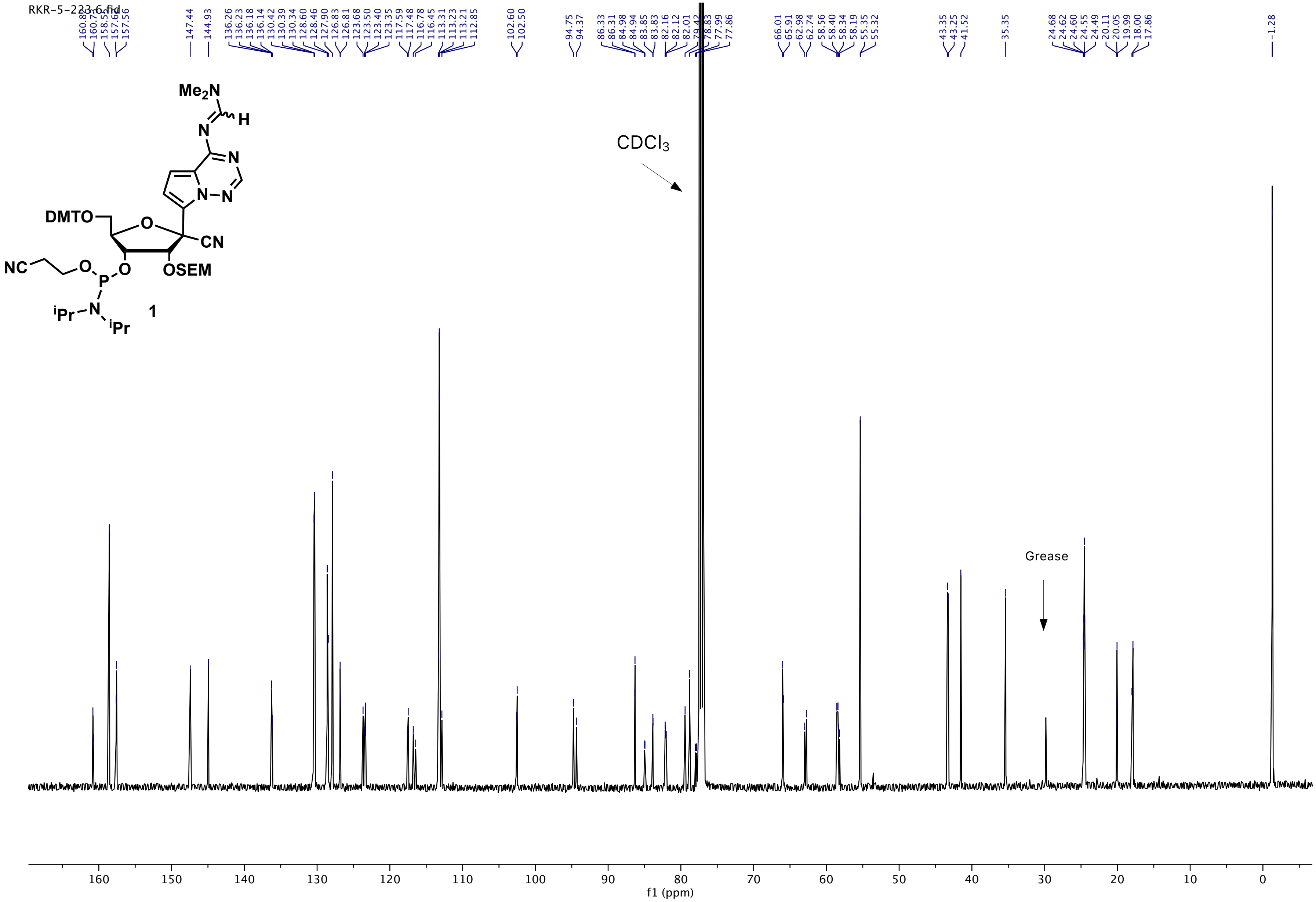
RKR-5-223.6.fid
Me$_2$N
H
N
DMTO
O
CN
NC
O
P
O
OSEM
^{i}Pr
N
^{i}Pr
1
CDCl$_3$
Grease
f1 (ppm)

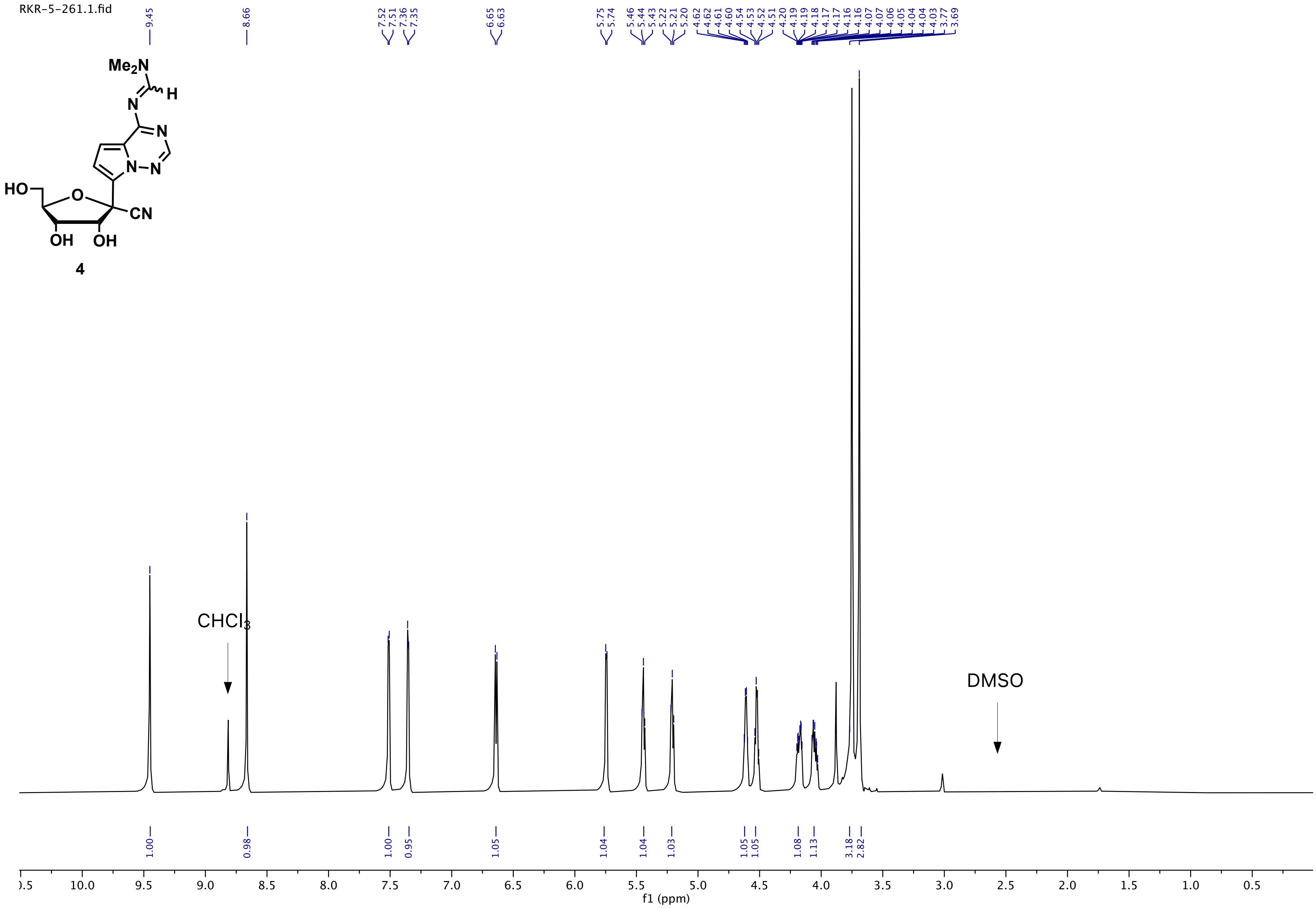
RKR-5-261.1.fid
9.45
8.66
7.52 7.51 7.36 7.35
6.65 6.63
5.75 5.74 5.46 5.44 5.43 5.22 5.21 5.20 4.62 4.62 4.61 4.60 4.54 4.53 4.52 4.51 4.20 4.19 4.19 4.18 4.17 4.17 4.16 4.16 4.07 4.07 4.06 4.05 4.04 4.04 4.03 3.77 3.69
Me2N
H
N
N
N
N–N
HO
O
CN
OH OH
4
CHCl3
DMSO
1.00 0.98 1.00 0.95 1.05 1.04 1.04 1.03 1.05 1.05 1.08 1.13 3.18 2.82
f1 (ppm)

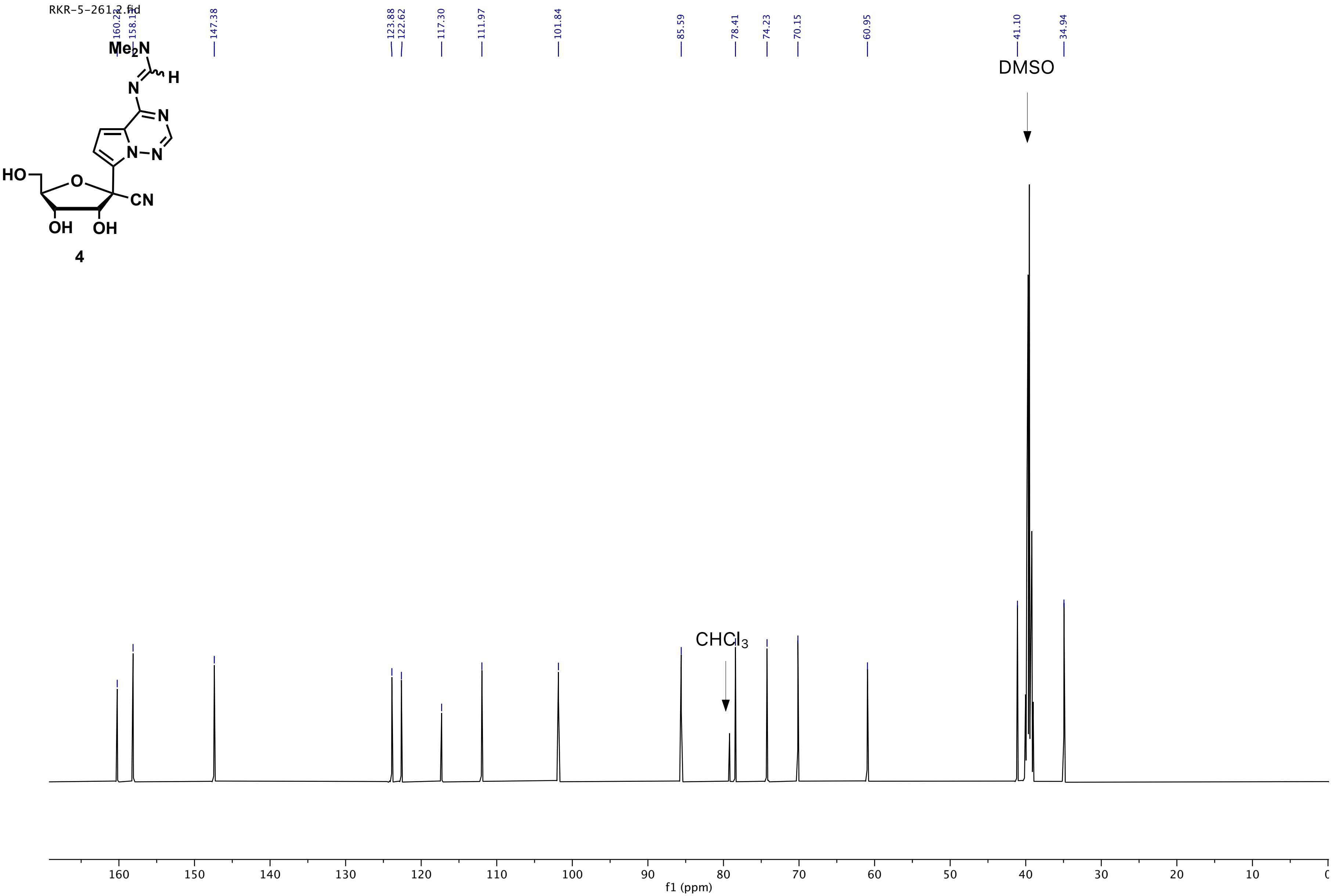
RKR-5-261.2.fid
160.2?
158.1?
147.38
123.88
122.62
117.30
111.97
101.84
85.59
78.41
74.23
70.15
60.95
41.10
34.94
DMSO
CHCl3
Me2N
H
N
N
N
N
N
HO
O
CN
OH
OH
4
160
150
140
130
120
110
100
90
80
70
60
50
40
30
20
10
f1 (ppm)

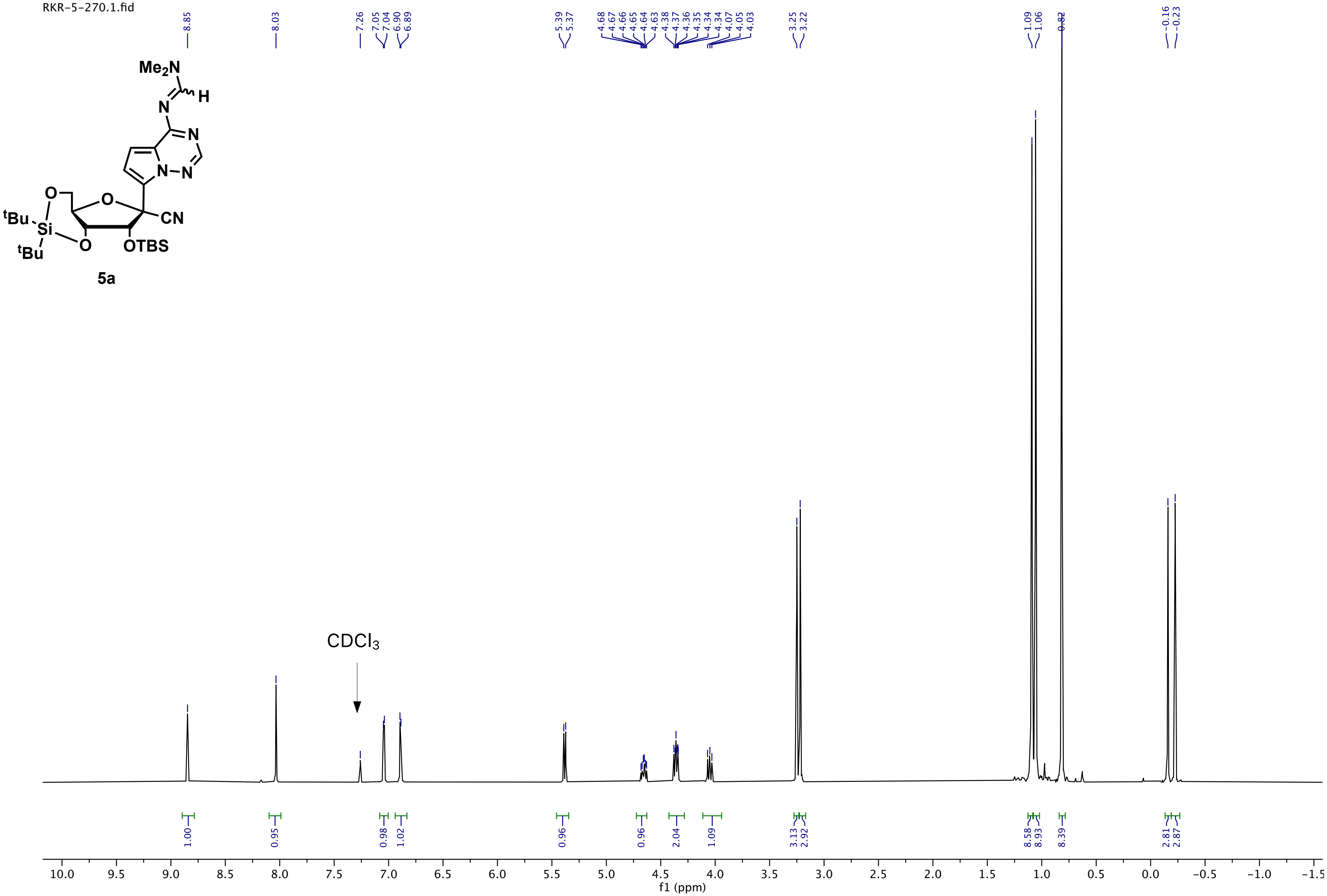
RKR-5-270.1.fid
Me2N
H
N
N
N
N
O
O
tBu
Si
tBu
O
CN
OTBS
8.85
8.03
7.26
7.05
7.04
6.90
6.89
5.39
5.37
4.68
4.67
4.66
4.65
4.64
4.63
4.38
4.37
4.36
4.35
4.34
4.34
4.07
4.05
4.03
3.25
3.22
1.09
1.06
0.82
-0.16
-0.23
CDCl3
1.00
0.95
0.98
1.02
0.96
0.96
2.04
1.09
3.13
2.92
8.58
8.93
8.39
2.81
2.87
10.0
9.5
9.0
8.5
8.0
7.5
7.0
6.5
6.0
5.5
5.0
4.5
4.0
3.5
3.0
2.5
2.0
1.5
1.0
0.5
0.0
-0.5
-1.0
-1.5
f1 (ppm)

5a

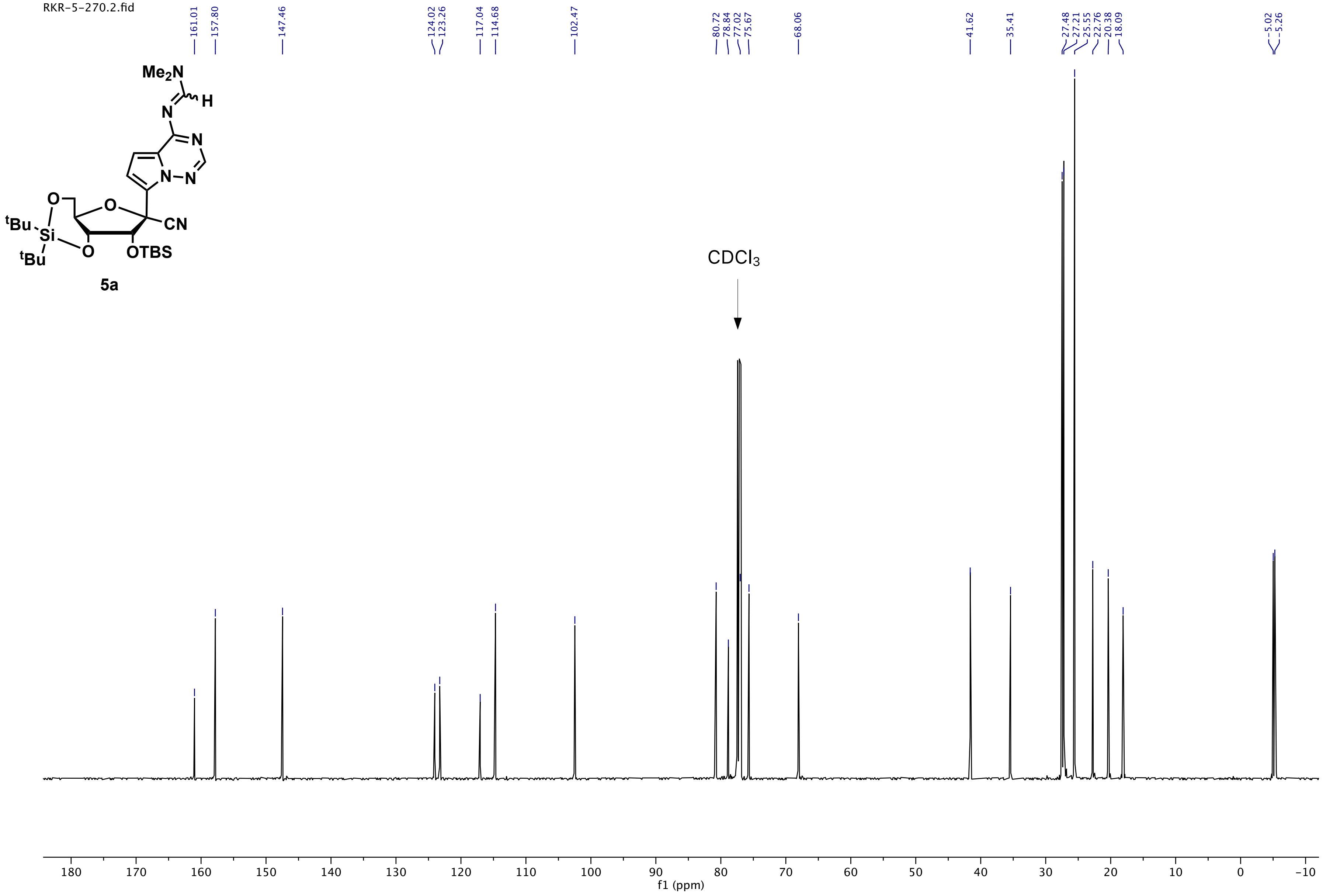

RKR-5-270.2.fid
161.01
157.80
147.46
124.02
123.26
117.04
114.68
102.47
80.72
78.84
77.02
75.67
68.06
41.62
35.41
27.48
27.21
25.55
22.76
20.38
18.09
-5.02
-5.26
Me_2N
H
N
O
CN
^tBu
Si
OTBS
5a
$CDCl_3$
f1 (ppm)

VII. LC-MS Trace and Mass Spectra of RNA-1.

RNA-1: 5′—CCGGGCGGCR—3′

Purity – 91% (monitored at 260 nm); MS calculated (*m/z* + Na^+): 3234.9; found (*m/z* + Na^+): 3238.0

HPLC Trace of ***RNA-1***

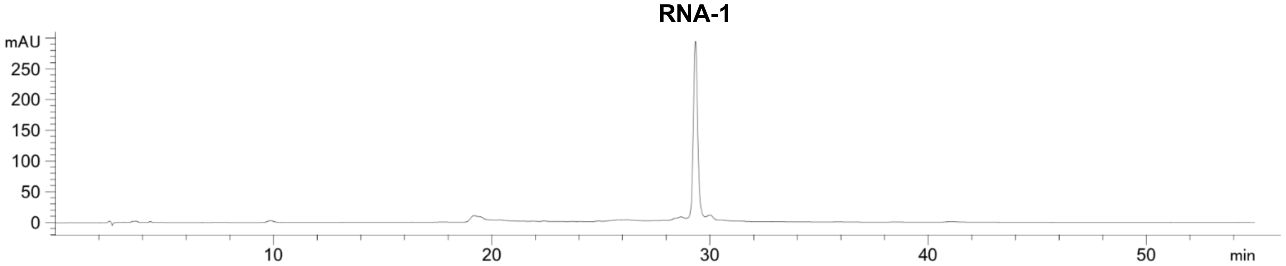

Mass Spectrometry Analysis of ***RNA-1***

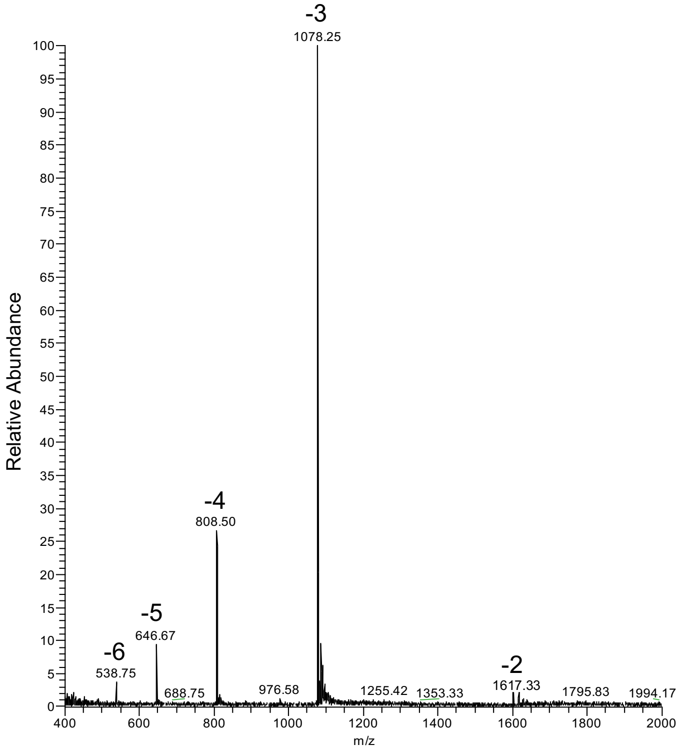

VIII. References.

1. Reddy, M. P., Hanna, N. B., Farooqui, F. *Tetrahedron Lett.*, **1994**, *35*, 4311-4314.
2. Wincott, F., DiRenzo, A., Shaffer, C., Grimm, S., Tracz, D., Workman, C., Sweedler, D., Gonzalez, C., Scaringe, S., Usman, N. *Nucleic Acids Res.*, **1995**, *23*, 2677-2684.
3. Sproat, B., Colonna, F., Mullah, B., Tsou, D., Andrus, A., Hampel, A., Vinayak, R. *Nucleosides Nucleotides*, **1995**, *14*, 255-273.
4. Pitsch, S., Weiss, P. A., Jenny, L., Stutz, A., Wu, X. *Helv. Chim. Acta*, **2001**, *84*, 3773-3795.
5. Kokic, G., Hillen, H. S., Tegunov, D., Dienemann, C., Seitz, F., Schmitzova, J., Farnung, L., Siewert, A., Hobartner, C., Cramer, P. *Nat. Commun.*, **2021**, *12*, 1-7.
